# Simultaneous Reversal of T Lymphocytes and Cancer Cells Metabolism Via a Biomimetic Heavy‐Atom‐Free Photosensitizers‐Based Combination Therapies to Boost Cancer Photoimmunotherapy

**DOI:** 10.1002/advs.202416143

**Published:** 2025-03-05

**Authors:** Yongjian Zhang, Xiaohong Wu, Kaiyuan Wang, Yaohan Tang, Xiuxin Lu, Fusheng Sun, Hua Tang, Xiaoyuan Chen, Shipeng Ning

**Affiliations:** ^1^ The Sixth Affiliated Hospital of Harbin Medical University Harbin Heilongjiang 150000 P. R. China; ^2^ The Fourth Affiliated Hospital of Harbin Medical University, NHC and CAMS Key Laboratory of Molecular Probe and Targeted Theranostics Harbin Medical University Harbin Heilongjiang 150001 P. R. China; ^3^ Department of Pharmaceutics Wuya College of Innovation Shenyang Pharmaceutical University Shenyang Liaoning 110016 P. R. China; ^4^ Departments of Diagnostic Radiology Surgery Chemical and Biomolecular Engineering and Biomedical Engineering Yong Loo Lin School of Medicine and College of Design and Engineering National University of Singapore Singapore 119074 Singapore; ^5^ Research Center of Nanomedicine Technology The Second Affiliated Hospital of Guangxi Medical University Nanning 530000 P. R. China; ^6^ Clinical Imaging Research Centre Centre for Translational Medicine Yong Loo Lin School of Medicine National University of Singapore Singapore 117599 Singapore; ^7^ Nanomedicine Translational Research Program Yong Loo Lin School of Medicine National University of Singapore Singapore 117597 Singapore; ^8^ Theranostics Center of Excellence (TCE) Yong Loo Lin School of Medicine National University of Singapore 11 Biopolis Way Helios 138667 Singapore; ^9^ Agency for Science Technology and Research (A*STAR) Institute of Molecular and Cell Biology Proteos, 61 Biopolis Drive Singapore 138673 Singapore; ^10^ Department of Pharmacy and Pharmaceutical Sciences National University of Singapore Lower Kent Ridge Road, 4 Science Drive 2 Singapore 117544 Singapore

**Keywords:** heavy‐atom‐free photosensitizer, lithium carbonate, NIR activated type I/II PDT, photoimmunotherapy, T lymphocytes and cancer cells metabolism

## Abstract

Near‐infrared (NIR) activated photosensitizers based on heavy‐atom‐free have great advantages in photoimmunotherapy, yet the tumor microenvironment often restricts their efficacy. To address this, a NIR‐activated heavy‐atom‐free photosensitizer (named Cy‐BF) is developed. Cy‐BF is then encapsulated with phospholipids and platelet exosome vesicles to create platelet exosomes vesicles biomimetic and Cy‐BF loaded hybrid liposomes (named CHL) Characterized by high phototoxicity, low dark toxicity, and enhanced tumor targeting, CHL demonstrates aggregation‐induced broadening of absorption spectra and NIR (760 nm laser) activates photothermal therapy and type I photodynamic therapy. The CHL‐mediated phototherapy induces mitochondrial damage and immunogenic cell death in tumor cells, decreases lactate production, and alters the tumor microenvironment by reducing regulatory T cells and increasing CD8^+^ T cells. To mitigate T cell inhibition by excess lactate, a combination therapy is introduced using lithium carbonate, which repurposes lactate as an energy source for CD8^+^ T cells, thereby enhancing the effectiveness of CHL‐mediated photoimmunotherapy. This combination approach represents a novel strategy for reversing lactate metabolism in both tumor cells and T cells, paving the way for future clinical applications in photoimmunotherapy.

## Graphical abstract

This work develops a cyanine dye named Cy‐BF and encapsulates it with phospholipids and platelet exosome vesicles to create CHL. Characterized by high phototoxicity, low dark toxicity, and enhanced tumor targeting, CHL demonstrates aggregation‐induced broadening of absorption spectra and NIR‐activated photothermal therapy and type I photodynamic therapy. This CHL‐mediated phototherapy induces immunogenic cell death in tumor cells, decreases lactate production, and alters the tumor microenvironment by reducing Tregs and increasing CD8+ T cells. Then a combination therapy using lithium carbonate (LC), which repurposes lactate as an energy source for CD8+ T cells, is used for enhancing CHL‐mediated photoimmunotherapy. This combination approach represents a novel strategy for reversing lactate metabolism in both tumor cells and T cells.

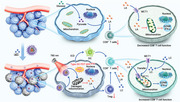

## 1 Introduction

Cancer remains a pervasive and deadly disease worldwide, presenting significant challenges to public health due to its high prevalence and mortality rates.^[^ ^1^ ^]^ Phototherapy, as a non‐invasive treatment method, has shown immense potential in the field of tumor treatment.^[^ ^2^ ^]^ Notable for its efficiency, selectivity, and low incidence of drug resistance, phototherapy is quickly becoming a focal point of research. This approach primarily encompasses photothermal therapy (PTT) and photodynamic therapy (PDT).^[^ ^3^ ^]^ During these treatments, photosensitizers (PSs) are activated by specific laser wavelengths to either generate heat or produce reactive oxygen species (ROS), both of which are effective in destroying tumor cells and tissues.^[^ ^4^ ^]^ The integration of phototherapy with immunotherapy, which leverages various immunological techniques to prompt the immune system to detect and eliminate tumor cells, offers a potent treatment for both primary and metastatic tumors.^[^ ^5^ ^]^ This strategy, known as photoimmunotherapy (PIT), utilizes the targeted destruction of primary tumors through light exposure while simultaneously triggering immune responses by promoting immunogenic cancer cell death.^[^ ^6^ ^]^ Therefore, by combining these therapies, PIT provides a novel anti‐cancer strategy that not only enhances the effectiveness of each treatment but also addresses their individual limitations.

Traditional photosensitizers typically use heavy metals because they can generate singlet oxygen (^1^O_2_) or other reactive oxygen species through effective electron and energy transfer mechanisms.^[^ ^7^ ^]^ Heavy‐atom‐free photosensitizers (HAF PSs) are those that exclude heavy metals like platinum, iridium, ruthenium, etc.^[^ ^8^ ^]^ Many HAF PSs are based on organic compounds and can degrade in the environment, reducing environmental pollution.^[^ ^8^, ^9^ ^]^ The benefits of using HAF PSs include their low toxicity in the absence of light, prolonged triplet lifetimes, strong photostability, and relatively low cost.^[^ ^10^ ^]^ Thus, there is a pressing need to develop new anti‐tumor immunotherapy systems utilizing HAF PSs.

Although phototherapy can activate anti‐tumor immunity, tumor cell metabolic reprogramming to shape a specific tumor microenvironment can inhibit the PIT effect.^[^ ^11^ ^]^ Lactate (LA), a primary byproduct of tumor glycolysis, is produced by tumor cells at rates up to 40 times higher than normal cells, with concentrations reaching 40 µmol g^−1^ within the tumor environment.^[^ ^12^ ^]^ Tumor‐infiltrating lymphocytes (TILs) are submerged in this lactate‐rich fluid, where LA transport into the cytoplasm of TILs can inhibit T cell function and activation.^[^ ^13^ ^]^ Additionally, lactate undermines immunotherapy effectiveness by aiding tumor DNA repair and increasing the presence of tumor‐associated macrophages (TAMs), myeloid‐derived suppressor cells (MDSCs), and regulatory T cells (Tregs).^[^ ^14^ ^]^ Recent studies have shown that lithium carbonate (LC, an emotion stabilizer) that reduces lactate levels can reverse lactate‐mediated CD8^+^ T cell immune suppression.^[^ ^12^ ^]^ LC inhibits lysosomal acidification and facilitates the movement of the monocarboxylate transporter 1 (MCT1) to the mitochondrial membrane, enabling lactate to be used as an energy source for CD8^+^ T cells. This innovative approach converts a waste product into a valuable resource, enhancing CD8^+^ T cell activity and overcoming lactate‐induced immune suppression. Consequently, integrating PIT with LC to manipulate tumor lactate metabolism could enhance the immune environment within tumors while repurposing lactate as an energy source for T cells, offering a promising avenue for effective tumor immunotherapy. This novel strategy for modulating lactate metabolism in cancer treatment is still underexplored in existing literature.

**Scheme 1:**
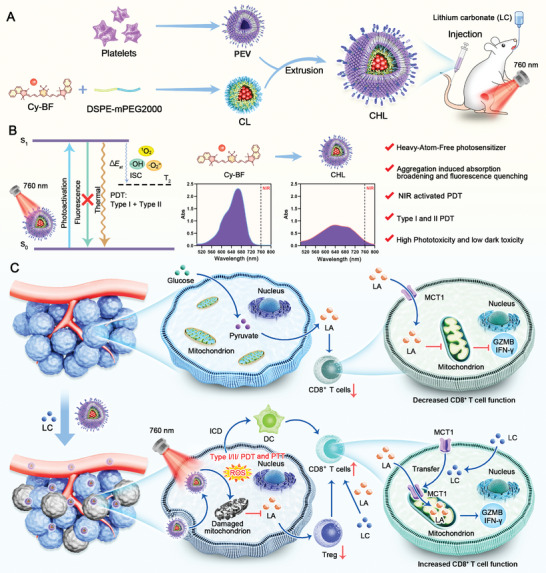
Schematic illustration of Biomimetic heavy‐atom‐free photosensitizers collaborate with lithium carbonate reverses T lymphocytes and cancer cells metabolism to enhance near‐infrared activated photoimmunotherapy. A) The preparation process of CHL and combination therapy administration mode. B) Photophysical mechanism and advantages of the CHL. C) The in vivo mechanism of CHL combined with LC in anti‐tumor treatment. In untreated tumor tissue, the production of LA by tumor cells through glycolysis inhibits the mitochondrial function of CD8^+^ T cells, thereby suppressing the production of granzyme and cytokines in T cells. After combined treatment with CHL and LC, on the one hand, CHL‐mediated PDT and PTT induced tumor cell death and mitochondrial damage, reducing their LA production. On the other hand, LC reversed the immunosuppressive effect of LA on CD8^+^ T cells and instead enhanced the killing ability of CD8^+^T cells. This dual strategy ultimately leads to a systemic anti‐tumor immune response.

Here, we have prepared a novel near‐infrared (NIR) activated HAF named Cy‐BF through molecular engineering. This photosensitizer was encapsulated with phospholipids (DSPE‐PEG2000) and platelet exosome vesicles (PEV) to create aggregated nanoparticles, referred to as CHL. Following administration, CHL was activated by 760 nm NIR laser irradiation for phototherapy. CHL possesses several advantageous features:1) Due to the absence of heavy atoms, CHL exhibits high phototoxicity with minimal dark toxicity; 2) CHL demonstrates aggregation‐induced broadening of absorption spectra, facilitating NIR PDT; 3) CHL combines Type I PDT capabilities to overcome tumor hypoxia; 4) Its aggregation‐induced fluorescence quenching enhances the conversion of light energy into heat and ROS and 5) platelet exosome vesicles (PEV) coating provide effective tumor targeting ability. Compared to other biomimetic carriers such as red blood cell exosomes (REV) and tumor‐derived exosomes (TEV), PEV has better advantages.^[^ ^15^ ^]^ Since PEV originates from blood, their biological safety is better than TEV. In addition, the PEV surface contains P‐selectin protein targeting tumors, which has excellent tumor‐targeting ability.^[^ ^5c^ ^]^ Furthermore, the platelet content in the blood is high and easy to obtain, making it one of the best candidates as a drug carrier. NIR laser is less absorbed by biological tissues within the wavelength range of 700–1700 nm, allowing for deeper penetration of tissues.^[^ ^16^ ^]^ CHL solves the problems such as laser penetration depth and oxygen dependence of traditional photosensitizers. During treatment with CHL, mice also received oral lithium carbonate (LC) therapy, which transformed lactate, a byproduct of tumor metabolism, into a valuable energy source for CD8^+^ T cells, thus enhancing the efficacy of CHL‐mediated photoimmunotherapy. This approach not only caused mitochondrial damage and immunogenic cell death in tumor cells but also altered the tumor microenvironment by reducing Tregs and increasing CD8^+^ T cells. Furthermore, this combination therapy effectively suppressed distant tumor growth by targeting primary tumors and maintained its efficacy even when exogenous lactate was administered to tumor‐bearing mice. This combination therapy reversed lactate metabolism in both tumor cells and T cells for the first time, providing an innovative approach for future clinical photoimmunotherapy (**Scheme** **1**).

## 2 Results and Discussion

### 2.1 Preparation and Characterization of Cy‐BF and CHL

**Figure 1:**
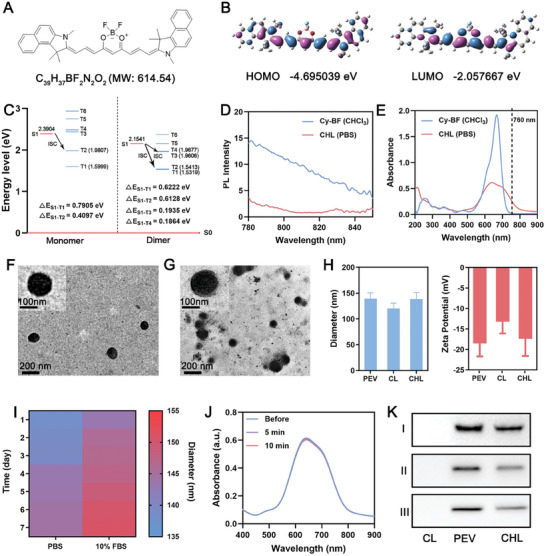
A) The chemical structural formula of Cy‐BF. B) HOMO‐LUMO distribution determined by DFT calculations for Cy‐BF. C) Energy levels for simplified monomeric and dimeric Cy‐BF at the optimized molecular geometries. D) Photoluminescence (PL) spectroscopy and E) absorption spectra of Cy‐BF in CHCl_3_ solution compared to CHL in PBS solution, using an excitation wavelength of 760 nm. F) TEM image of CL and G) CHL, including a magnified view of a particle. H) Particle diameter and Zeta potential measurements for PEV, CL, and CHL. Data are shown as the mean ± SD (*n* = 3). I) Stability of CHL in various solutions. J) Absorption spectra of CHL before and after exposure to 760 nm laser irradiation at 0.5W/cm^2^ for varying durations. K) Western blot (WB) analysis of key proteins in different formulations (I: Actin, II: P‐selectin, III: CD41) at a Cy‐BF concentration of 10 µg mL^−1^.

First, we designed a novel small molecule‐based cyanine boron fluoride derivative photosensitizer named Cy‐BF. The synthesis route, molecular structure, High‐Resolution Mass Spectrometry (HRMS), and ^1^H Nuclear Magnetic Resonance (NMR) spectrum of Cy‐BF were displayed in Scheme S1 (Supporting Information), **Figure** **1**A and Figures S1 and S2 (Supporting Information) respectively, confirming the successful synthesis of these compounds. The standard curve of 660 nm Cy‐BF is shown in Figure S3 (Supporting Information). To understand the detailed mechanism of triplet state formation, we performed theoretical calculations on the Cy‐BF structures using time‐dependent density functional theory (TD‐DFT). As illustrated in Figure 1B, the highest occupied molecular orbital (HOMO) of Cy‐BF spans the entire molecular framework, while the lowest unoccupied molecular orbital (LUMO) is predominantly localized in the central acceptor region. The triplet state (T1−S0) energy gaps for Cy‐BF were calculated to be 1.599 eV, which were higher than those required to excite ^3^O_2_ for ^1^O_2_ (0.977 eV) via energy transfer.^[^ ^17^ ^]^ Similarly, the ΔE_S1−T1_ value was 0.7905 eV, which is too large to facilitate intersystem crossing (ISC). However, the ΔE_S1−T2_ was significantly reduced to 0.4079 eV, suggesting a viable ISC pathway from the S1 to the T2 state (Figure 1C), thereby enhancing ROS generation. The energy gap (Δ_EST_) between the first excited singlet state (S1) and the first excited triplet state (T1) of dimeric Cy‐BF (Figure S4, 0.6222 eV) was smaller than that of the monomer (0.7905 eV), and the Tn (n > 1) energy levels of the dimer are significantly decreased. With the increase of polymerization degree, the energy level difference between the excited states is gradually approaching, which provides more feasible pathways for inter‐system transition from singlet to triplet states, thereby facilitating inter‐system transition and improving the efficiency of singlet oxygen production.^[^ ^18^ ^]^ Subsequently, Cy‐BF (referred to as CL) was encapsulated with DSPE‐PEG2000, and the platelet exosome vesicles (PEV) were physically extruded onto the surface of CL to form CHL. Fluorescence spectroscopy showed that Cy‐BF in chloroform displayed strong fluorescence under 660 or 760 nm light, but this fluorescence was significantly quenched in the CHL format (Figure 1D; Figure S5, Supporting Information). The quenching of fluorescence indicates that upon aggregation, Cy‐BF efficiently converts light energy into heat and demonstrates robust ISC capabilities, enhancing the effectiveness of PDT. As shown in Figure 1E, at the same concentration, while the absorption peak of CHL at the same concentration was lower than that of Cy‐BF, there was a noticeable broadening of the absorption spectrum and a double peak occurrence. This could be likely due to the M‐aggregation of Cy‐BF in CHL.^[^ ^19^ ^]^ The absence of a red or blue shift in the CHL spectrum indicates that it does not exhibit either H or J‐aggregation.^[^ ^20^ ^]^ This also significantly increased the near‐infrared (NIR) absorption capacity of Cy‐BF, facilitating its activation by NIR laser for both PDT. The molar extinction coefficients of Cy‐BF and CHL at 760 nm were 1.25 × 10^3^ L mol^−1^ cm^−1^ and 1.40 × 10^4^ L mol^−1^ cm^−1^, respectively, indicating that CHL has improved 760 nm light‐harvesting ability. TEM images of CL, PEV, and CHL have been shown in Figures 1F,G and S6 (Supporting Information). There was an obvious membrane structure on the surface of CHL, with a particle size ≈10 nm higher than CL, and a zeta potential like PEV (Figure 1H), confirming that PEV has effectively enveloped the surface of CL. The drug loading efficiency of Cy‐BF in CHL is to be 37.4 ± 5.2% through absorption spectroscopy and standard curves. CHL demonstrated good dimensional stability (Figure 1I) and photostability (Figure 1J), and the surface contains platelet membrane intrinsic proteins CD41 and P‐selectin (Figure 1K). P‐selectin plays a critical role in targeting the CD44 receptor on tumor surfaces.^[^ ^21^ ^]^ Compared to CL, CHL exhibited better tumor‐targeting ability (Figure S7, Supporting Information). In summary, we have successfully synthesized CHL, which has excellent NIR absorption, tumor targeting, and photostability, laying the foundation for subsequent biological experiments.

### 2.2 Photothermal Ability, ROS Generation, and In Vitro Anticancer Ability of CHL

**Figure 2:**
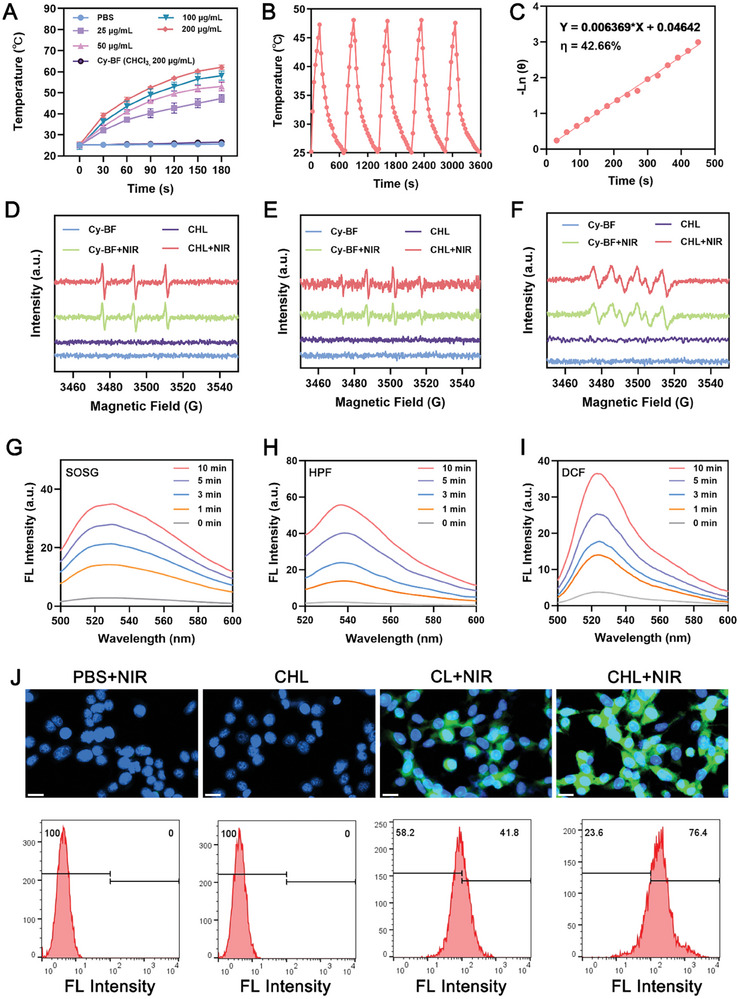
A) Heating profile of CHL containing different concentrations of Cy‐BF, PBS, and chloroform solution containing 200 ug mL^−1^ Cy‐BF under 760 nm laser irradiation (0.5W/cm^2^) for 3 min. Data are shown as the mean ± SD (*n* = 3). B) Photothermal cycling curves of different formulations. C) Calculation of the time constant for heat transfer using a linear regression of the CHL cooling profile. D) Production of ^1^O_2_, E) •OH and F) •O_2_^−^ by the CHL or CHL+NIR (0.5 W cm^−2^, 3 min) was determined by ESR. G) Fluorescence spectra of SOSG, H) HPF, and I) DCF after CHL plus 760 nm laser irradiation (0.5 W cm^−2^) for different times. J) Fluorescence images and flow cytometry analysis of intracellular ROS treated with different components (NIR: 760 nm laser, 0.5 W cm^−2^, 10 min). Scale bars: 20 µm.

Next, we measured the photothermal performance of CHL, as shown in **Figure** **2**A–C. As the concentration increases, CHL heats up faster under near‐infrared light. CHL has stable photothermal cycling ability and high photothermal conversion efficiency. Electron spin resonance (ESR) spectroscopy was used to detect ROS production in Cy‐BF and CHL. As shown in Figure 2D–F, both CHL and Cy‐BF can produce ^1^O_2_, OH, and •O_2_^−^, with CHL producing more ROS than Cy‐BF at equivalent concentrations. We detected the production of ROS by CHL at different laser exposure times using different ROS indicators (as shown in Figure 2G–I; Figure S8, Supporting Information). These experimental results suggested that CHL can perform both type I and type II PDT simultaneously under 760 nm laser irradiation. According to previous studies, photosensitizers facilitating type I PDT are particularly effective in treating hypoxic tumor environments, highlighting the potential of CHL as a promising HAF photosensitizer. In addition, CHL can perform NIR‐activated PDT, which is crucial for advanced photosensitizers. Due to the issue of laser penetration ability, traditional visible light excited photosensitizers cannot penetrate deep tissues, which is not conducive to clinical translation.^[^ ^22^ ^]^ The development of NIR‐excited photosensitizers has always been a hot topic in the field of phototherapy research.^[^ ^16^, ^23^ ^]^

**Figure 3:**
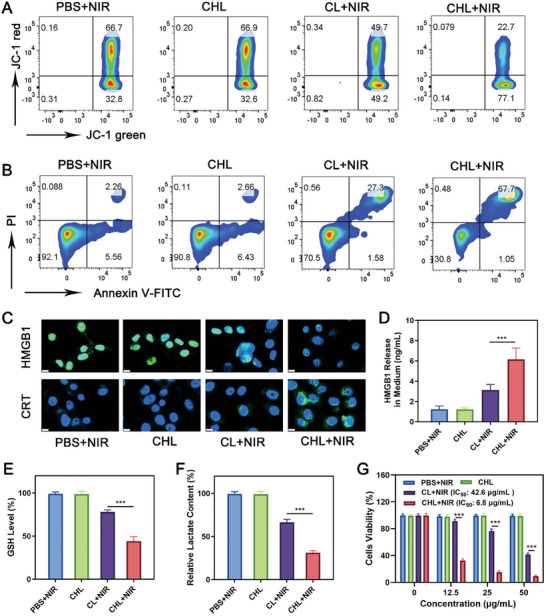
A) JC‐1 flow cytometry analysis and B) apoptosis detection of 4T1 cells treated with different components (NIR: 760 nm laser, 0.5 W cm^−2^, 10 min). C) Expression of HMGB1 and CRT (scale bars: 10 µm) and D) HMGB1 release from 4T1 cells following treatment with different formulations (NIR: 760 nm laser, 0.5 W cm^−2^, 10 min). E) Intracellular GSH and F) lactate levels after different treatments. Cy‐BF concentration: 100 µg mL^−1^. G) The relative cellular viability of 4T1 cells after various treatments with different Cy‐BF concentrations. NIR: 760 nm laser, 0.5 W cm^−2^, 10 min. Data are shown as the mean ± SD (*n* = 3). Statistical significance was calculated via one‐way ANOVA with Tukey's test: ^***^ *p* < 0.001.

**Figure 4:**
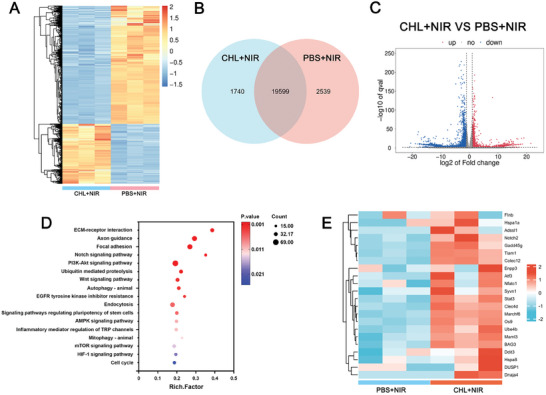
A) Heatmap illustrating differentially expressed genes after treatment. B) The Venn diagram revealed the number of genes transcribed in the indicated treatment group. C) Volcano plots illustrating differentially expressed genes. D) Results of KEGG pathway enrichment analysis. E) Heatmap of gene expressions related to apoptosis, immunity, and oxidative stress in 4T1 cells treated with PBS + NIR and CHL + NIR.NIR: 760 nm laser, 0.5 W cm^−2^, 10 min.

We next assessed the cytotoxic impact of CHL on 4T1 cells. As shown in Figures 2J,A and S9 (Supporting Information), CHL+NIR treatment led to an increase in ROS and altered mitochondrial membrane potential in 4T1 cells, compared to PBS+NIR, CHL, and CL+NIR groups. TEM results showed a decrease in mitochondrial cristae in tumor cells in the CHL+NIR group (Figure S10, Supporting Information), suggesting mitochondrial damage induced by CHL+NIR. CHL+NIR treatment also led to more cell apoptosis and immunogenic cell death (ICD, **Figure** **3**B–D: Figures S11–S13, Supporting Information). Immunofluorescence staining for ICD markers (HMGB1 and calreticulin, CRT) showed that cells treated with CHL and exposed to 760 nm laser radiation displayed markedly reduced green fluorescence in the nucleus (indicative of HMGB1 release) and increased green fluorescence on the cell membrane (indicative of elevated CRT expression). The release of HMGB1 and ATP from cells in the CHL+NIR group also significantly increased. These results suggest that the oxidative stress triggered by CHL+NIR can cause ICD of 4T1 cells. CHL+NIR treatment also resulted in decreased glutathione (GSH) and lactate (LA) levels in 4T1 cells (Figure 3E,F). The CCK‐8 assay corroborated the cytotoxic effects of CHL+NIR on 4T1 cells (Figure 3G). The results of transcriptomic sequencing illustrated that differential genes were significantly enriched in signaling pathways related to ICD, ROS, and immune regulation, further proving the potential of CHL in immunosensitization therapy (**Figure** **4**A–E). Relative to the PBS+NIR group, CHL+NIR treatment upregulated 1059 genes and 2263 transcripts while downregulating 2085 genes and 4053 transcripts (Figure S14, Supporting Information). For instance, the transcription levels of ATF3, HSPA1A, DDIT3, and HSPA8, related to ROS‐induced ER stress, were upregulated, likely due to ICD.^[^ ^24^ ^]^ Elevated transcription levels of GADD45G, NOTCH2, BAG3, and DUSP1 linked to apoptosis,^[^ ^25^ ^]^ indicated profound apoptosis triggered by CHL+NIR. Further KEGG pathway enrichment analysis indicated significant impacts on genes associated with ICD and apoptosis due to CHL+NIR treatment. As shown in Figure S15 (Supporting Information), under hypoxic conditions, the anti‐tumor ability of CL is not affected by oxygen. CL has better anti‐tumor effects compared to ICG‐DSPE. This is because Cy‐BF can generate hydroxyl radicals through type‐I PDT, which does not consume oxygen. Therefore, CL is less oxygen‐dependent compared to traditional photosensitizers such as ICG. And there is a hypoxic microenvironment in tumor tissue,^[^ ^26^ ^]^ so Cy‐BF has greater advantages. Under no‐laser conditions, CHL showed minimal cytotoxic effects on RAW 264.7 mouse macrophages and Cy‐BF showed minimal cytotoxic effects on 4T1 cells (Figures S16 and S17, Supporting Information), indicating its low dark toxicity and favorable biocompatibility.

**Figure 5:**
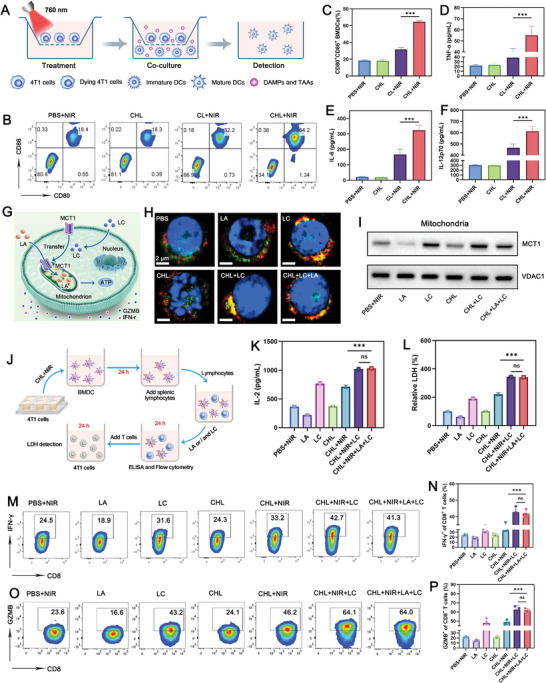
A) Schematic illustration of the Transwell system of the Transwell system used to induce maturation of BMDCs in vitro. B) Flow cytometry analysis and C) quantitative analysis of mature BMDCs after different treatments. The Cy‐BF concentration was 20 µg mL^−1^. Data are shown as the mean ± SD (*n* = 3). D) The levels of TNF‐α, E) IL‐6, and F) IL‐12p70 secreted by matured BMDCs. G) A schematic showing how LC facilitates the translocation of MCT1 from the T cell membrane to mitochondria. H) Immunofluorescence staining of MCT1 and COX IV in CD8^+^ T cells treated with indicated formulations. Blue: Nuclei, Green: COX IV and Red: MCT1. I) Western blot of MCT1 in the mitochondria of CD8^+^ T cells treated with indicated formulations. J) A schematic representing specific immune activation experimental setups in vitro. K) Detection of IL‐2 in supernatant from treated CD8*^+^* T cells by ELISA after LA or/and LC for 24h. L) Measurement of LDH levels in the supernatant after co‐incubation of the splenic T lymphocytes with 4T1 tumor cells for 24h. M) Representative plots and N) quantification of IFN‐γ in CD8^+^ T cells after different treatments analyzed by the flow cytometry. O) Representative plots and P) quantification of granzyme B (GZMB) in CD8^+^ T cells after different treatments analyzed by flow cytometry. LA and LC were used at a concentration of 10 mM, and Cy‐BF at 100 µg mL^−1^ Data are shown as the mean ± SD (*n* = 3). Statistical significance was calculated via one‐way ANOVA with Tukey's test: ns: Non‐Significant, ^***^ *p* < 0.001.

Given that the release of damage‐associated molecular patterns (DAMPs) can stimulate dendritic cells (DCs), we assessed DC maturation by co‐incubating bone marrow‐derived dendritic cells (BMDCs) with 4T1 cells treated using different groups in a Transwell system for 24 h (**Figure** **5**A). The expression of costimulatory molecules CD80 and CD86 served as indicators of DC maturity (Figure 5B,C). Following the release of DAMPs, cancer cells treated with CHL+NIR notably enhanced BMDC maturation to 65.2%, a 1.97 fold increase compared to those treated with CL+NIR. This maturation was accompanied by a significant rise in proinflammatory cytokines such as IL‐12p70, TNF‐α, and IL‐6 compared to the control group (Figure 5D–F). Subsequently, we investigated the effects of LC combined with CHL on immune cells in vitro. As shown in Figure 5G, previous studies have shown that LC‐mediated transfer of MCT1 to the mitochondrial membrane allows lactate to enter the mitochondria as an energy source, thereby providing energy for CD8^+^ T cells.^[^ ^12^ ^]^ Using fluorescence microscopy and western blot analyses (Figure 5H,I; Figure S18, Supporting Information), we observed that after LC treatment, MCT1 protein in CD8^+^ T cells predominantly localized to the mitochondria, unaffected by CHL or LA treatments. As shown in Figure 5J, we established an assay based on BMDCs and splenic T lymphocytes in vitro. Flow cytometry was used to demonstrate that the CHL+NIR+LC group or CHL+NIR+LC+LA group can effectively activate T lymphocytes after activating BMDCs. This resulted in a significant increase in IL‐2 release and the expression of IFN‐γ and GZMB compared to the LA or PBS groups (Figure 5K,M–P). When these T lymphocytes were co‐cultured with 4T1 tumor cells for 24 h, lactate dehydrogenase (LDH) levels in the supernatant confirmed effective tumor cell killing by the CHL+NIR+LC and CHL+NIR+LC+LA treatment (Figure 5L). It is worth noting that LA treatment can significantly reduce T cell activation and anti‐tumor factor production, but the introduction of LC counteracted this effect, demonstrating that this combination therapy successfully overcomes lactate‐induced suppression of T cells.

### 2.3 In Vivo Anti‐Tumor Ability of CHL

**Figure 6:**
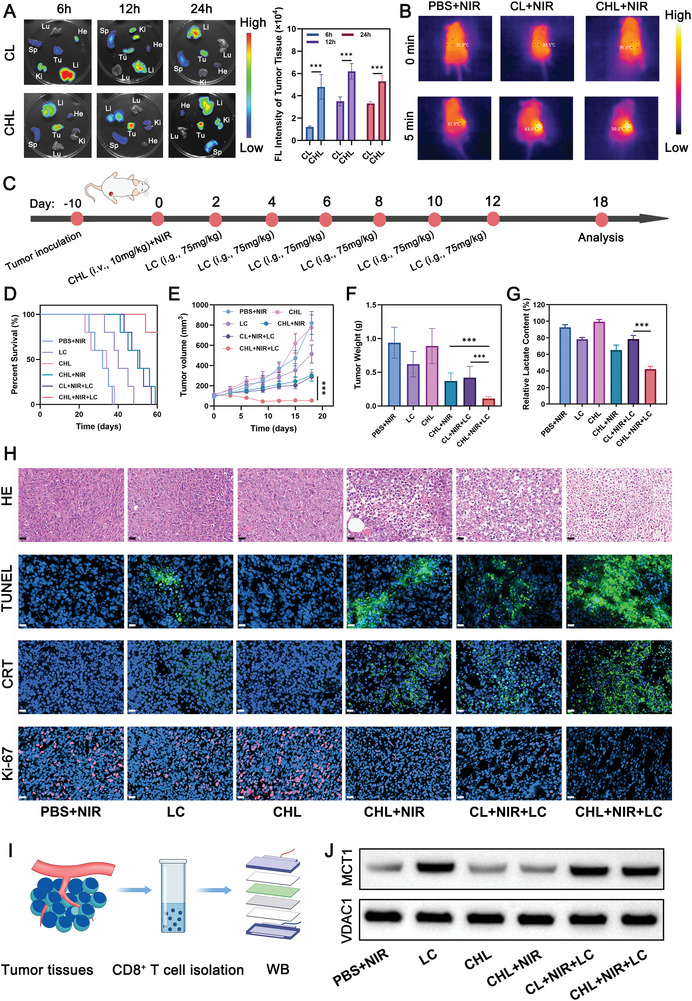
A) Ex vivo fluorescence imaging and corresponding intensity data of tumors and organs from 4T1 tumor‐bearing mice at various post‐injection intervals of CL or CHL. B) Infrared thermography of tumor tissue after different treatments. C) A schematic Diagram outlining the timeline for tumor inoculation, nanodrug administration, laser treatment, and monitoring of tumor growth. D) Survival curves after treatment. E) Tumor volumes were measured every 3 days across all groups. F) Tumor weights were recorded for each treatment at the end of the study. G) Relative lactate content in 4T1 tumor tissues after different treatments. H) H&E, TUNEL, CRT, and Ki‐67 staining of tumor sections after the indicated treatments. Scale bars: 20 µm. I) Schematic illustration of western blotting analysis of protein expression in CD8^+^ T cells. J) Western blotting analysis of VDAC1 and MCT1 expression in CD8^+^ T cell mitochondrion. Cy‐BF dose: 10 mg kg^−1^. Data are shown as the mean ± SD (*n* = 5). Statistical significance was calculated via one‐way ANOVA with Tukey's test: ^***^ *p* < 0.001.

**Figure 7:**
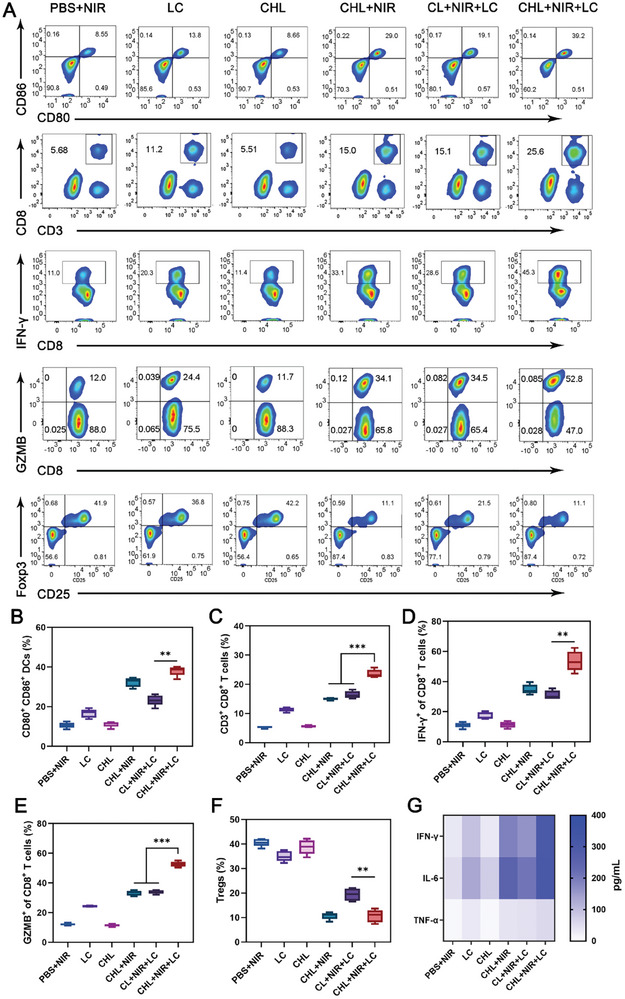
A) Flow cytometry data showing the effects of treatments on DCs maturation in lymph nodes, infiltration of CD3^+^ CD8^+^ T lymphocytes in 4T1 tumor tissues, and levels of IFN‐γ^+^ and GZMB^+^ cells among CD8^+^ T cells, as well as CD25^+^ Foxp3^+^ Tregs in tumor tumor tissues. B) Quantitative analysis of DCs maturation in lymph nodes induced by treatments, C) CD3^+^ CD8^+^ T lymphocytes infiltration in the 4T1 tumor tissues, D) IFN‐γ^+^ and E) GZMB^+^ cells among CD8^+^ T cells, and F) CD4^+^ Foxp3^+^ Tregs in tumor tissues. G) Secretion of pro‐inflammatory cytokines (TNF‐α, IFN‐γ, and IL‐6) in sera after exposure to different treatments. Data are shown as the mean ± SD (*n* = 5). Statistical significance was calculated via one‐way ANOVA with Tukey's test: ^**^ *p* < 0.01, ^***^ *p* < 0.001.

Building on the promising in vitro findings, we further explored the therapeutic potential of CHL‐mediated NIR photoimmunotherapy in combination with LC in an in vivo setting. BALB/c mice were subcutaneously injected with 5 × 10^6^ 4T1 cells into the right flank to form tumors. All animal procedures were performed in accordance with the guidelines for Care and Use of Laboratory Animals of the Ministry of Health in the People's Republic of PR China and approved by the Animal Ethics Committee of Guangxi Medical University (Approval number: 2023‐KY (0931)). As shown in **Figure** **6**A, CHL demonstrated superior tumor‐targeting capabilities compared to CL. Compared to the CL + NIR group, the CHL+NIR group reached a higher temperature at the tumor site after 10 min of illumination (Figure 6B). The flowchart of the CHL combined with the LC anti‐tumor experiment is shown in Figure 6C. LC was administered orally to mice every alternate day over a span of 10 days. CHL was intravenously injected through the tail vein on day 0 and phototherapy was performed 12 h later. The survival curve, tumor volume growth, and tumor weight at day 18 have been shown in Figure 6D–F. The data clearly indicates that the combination of CHL‐mediated phototherapy and LC treatment yielded the most significant anti‐tumor effects and enhanced survival rates. Furthermore, the lactate content in the tumor was notably lower in this group compared to control groups (Figure 6G), indicating effective inhibition of lactate production in tumor cells by this combination therapy. In vivo ROS staining indicated that the ROS levels induced by CHL+NIR or CHL+NIR+LC were higher than those induced by CL+NIR+LC, highlighting greater photodynamic PDT efficiency with CHL (Figure S19, Supporting Information). Histological examination of tumor tissues through hematoxylin‐eosin (HE) staining, immunofluorescence staining for CRT, HMGB1, and terminal‐deoxynucleotidyl transferase‐mediated nick end labeling (TUNEL) revealed extensive necrosis, ICD, and apoptosis in tumors treated with CHL+NIR+LC (Figure 6H; Figure S20, Supporting Information). Additionally, this treatment group exhibited a significant reduction in cell proliferation, as evidenced by lower Ki‐67 levels, indicating a substantial inhibition of tumor growth. We isolated CD8^+^ T cells from tumor tissue for western blot (WB) and flow cytometry analysis. As shown in Figure 6I,J and Figures S21 and S22 (Supporting Information), the expression of MCT1 in CD8^+^ T cell mitochondria significantly increased after LC treatment, aligning with our in vitro, thereby indicating LC‐mediated T cell membrane MCT1 transfer. The maturation of DCs in tumor‐draining lymph nodes markedly increased in the CHL+NIR+LC group, alongside a rise in the number of tumor‐infiltrating CD8^+^ T cells and the expression of IFN‐γ and GZMB in these cells (**Figure** **7**A–F), demonstrating effective activation of T cell‐mediated immunity. In addition, the number of Tregs in tumor tissue significantly decreased, thanks to the decrease in lactate content in tumor tissue. Moreover, the serum levels of key cytokines such as tumor necrosis factor‐alpha (TNF‐α), interleukin‐6 (IL‐6), and IFN‐γ were significantly elevated in the CHL+NIR+LC group, as measured by enzyme‐linked immunosorbent assay (ELISA) (Figure 7G), indicating a robust systemic anti‐tumor immune response. In addition, we compared the anti‐tumor ability of CL+NIR and CHL+NIR (as shown in Figure S23, Supporting Information) and found that CHL+NIR has better anti‐tumor ability than CL+NIR, possibly due to CHL's better tumor targeting ability. Finally, after treatment, no significant liver or kidney toxicity or weight loss was observed, confirming the good biocompatibility of the system (Figure S24, Supporting Information).

**Figure 8:**
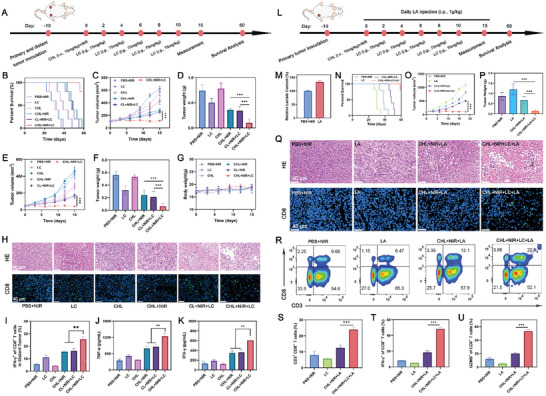
A) Schematic illustration of the studies of 4T1 bilateral tumor therapy. B) Survival curves after different treatments. C) Evolution of the primary tumor volume and D) tumor weight after various treatments. E) Development of distant tumor volume and F) tumor weight following various treatments. G) Changes in body weight during the treatment period. H) H&E and CD8 staining analyses of distant tumor tissues after different treatments. I) Quantification of treatment‐induced IFN‐γ^+^ cells among CD8^+^ T cells. J) Levels of pro‐inflammatory cytokines (TNF‐α and K) IFN‐γ) in serum following different treatments. L) Schematic illustration of the studies of 4T1 tumor therapy. M) Lactate content in tumor tissue after different treatments. N) Survival curves after different treatments. O) Changes in tumor volume and P) tumor weight after different treatments. Q) H&E and CD8 staining analyses of tumor tissues treated with various treatments. R) Flow cytometry analysis and S) Quantification of CD3^+^ CD8^+^ T lymphocytes infiltration in the 4T1 tumor tissues. T) Quantification of treatment‐induced IFN‐γ^+^ and U) GZMB^+^ cells among CD8^+^ T cells. Data are shown as the mean ± SD (*n* = 5). Statistical significance was calculated via one‐way ANOVA with Tukey's test: ^**^ *p* < 0.01, ^***^ *p* < 0.001.

An in vivo study on anti‐bilateral tumor effects was conducted through the systemic administration of various treatment formulations (**Figure** **8**A). The growth of primary and distant tumors was monitored every three days over a period of 15 days. Treatment regimens including free CL+NIR+LC, CHL+NIR, and CL+NIR+LC showed a relative delay in tumor growth compared to the PBS control group (Figure 8B–F). Remarkably, the CHL+NIR+LC treatment group significantly hindered the growth of both primary and distant tumors, achieving inhibition rates of 81.7% and 90.9%, respectively. This group also showed superior performance in reducing the tumor weights of both primary and distant tumors. In addition, no obvious weight loss of mice was observed after various treatments (Figure 8G). H&E staining revealed extensive nuclear disintegration and significant necrotic areas in distant tumors from the CHL+NIR, CL+NIR+LC, and CHL+NIR+LC groups post‐phototherapy, whereas no significant cell death was observed in tumors from other treatment groups (Figure 8H). After treatment with CHL+NIR+LC, there was a marked increase in the population of CD8^+^ T cells and the subsets expressing high levels of IFN‐γ in the distant tumor tissues (Figure 8I; Figure S25, Supporting Information). ELISA analysis revealed that combinatory therapy by CHL+NIR + LC dramatically induced sera IFN‐γ and TNF‐α (Figure 8J,K). Collectively, these results confirm that CHL‐mediated NIR PIT combined with LC effectively delays primary tumor growth and induces abscopal effects, thereby inhibiting the growth of distant tumors.

To further validate the effect of LA on this combination therapy, we administered exogenous LA via intraperitoneal injection to mice, as shown in Figure 8L. After intraperitoneal injection of LA, the amount of LA in tumor tissue increased (Figure 8M). The introduction of LA significantly diminished the therapeutic efficacy of the CHL+NIR group yet had minimal impact on the efficacy of the CHL+NIR+LC group (Figure 8N–P). The proportion of CD8^+^ T cells, GZMB^+^ CD8^+^, and IFN‐γ^+^ CD8^+^ T cells increased in the tumor tissues of the CHL+NIR+LC+LA group (Figure 8Q–U; Figure S26, Supporting Information). There was no notable change in the CHL+NIR+LC group compared to the first model8. Overall, the addition of LC mitigated the suppressive effects of tumor tissue lactate on T cells and enhanced the efficacy of CHL‐mediated PIT.

## 3 Conclusion

In conclusion, we have developed a novel NIR‐activated HAF photosensitizer named Cy‐BF, which was encapsulated with phospholipids and PEV to create CHL. CHL exhibits good tumor‐targeting capabilities and minimal toxicity in the absence of light. CHL plus NIR laser treatment leads to mitochondrial damage, lactate decrease, and immunogenic cell death in tumor cells. Additionally, CHL reduced regulatory T cells (Tregs) and increased CD8^+^ T cells within the tumor microenvironment. Then the integration of LC was able to transform lactate, allowing CD8^+^ T cells to utilize LA as an energy source, thus significantly boosting the effectiveness of CHL‐mediated photoimmunotherapy. This combination approach markedly inhibits the growth of distant tumors by treating primary tumors. Interestingly, while the administration of exogenous lactate to tumor‐bearing mice impaired phototherapy, it did not modulate the efficacy of the combined treatment. This innovative combination therapy, which can alter lactate metabolism in both tumor cells and T cells for the first time, holds tremendous potential for enhancing future clinical PIT. In the future, this combination therapy may increase the sensitivity of immune checkpoint inhibitor therapy and radiation immunotherapy. Furthermore, it may resolve the issues of hypoxia and T‐cell suppression that occur during radiotherapy.

## Conflict of Interest

The authors declare no conflict of interest.

## Supporting information



Supporting Information

## Data Availability

Data is available from the corresponding author upon reasonable request. Source data are provided with this paper.
