# Supplementary material for: Simultaneous Reversal of T Lymphocytes and Cancer Cells Metabolism Via a Biomimetic Heavy‐Atom‐Free Photosensitizers‐Based Combination Therapies to Boost Cancer Photoimmunotherapy
Source: Adv Sci (Weinh). 2025 Mar 5;12(16):2416143. doi: 10.1002/advs.202416143 (PMC12021059; doi:10.1002/advs.202416143)
Supplement: Supplementary file 1 — Supporting Information [file ADVS-12-2416143-s001.docx]

**Supporting Information**

**Simultaneous Reversal of T Lymphocytes and Cancer Cells Metabolism via a Biomimetic Heavy-atom-free Photosensitizers-based Combination Therapies to Boost Cancer Photoimmunotherapy**

Yongjian Zhang, Xiaohong Wu, Kaiyuan Wang*, Yaohan Tang, Xiuxin Lu, Fusheng Sun, Hua Tang, Xiaoyuan Chen*, Shipeng Ning*

Y. Zhang and F. Sun

The Sixth Affiliated Hospital of Harbin Medical University, Harbin, Heilongjiang, 150000, China.

X. Wu

Harbin Medical University, NHC and CAMS Key Laboratory of Molecular Probe and Targeted Theranostics, Harbin, Heilongjiang, 150000, China.

K. Wang

Department of Pharmaceutics, Wuya College of Innovation, Shenyang Pharmaceutical University, Shenyang, Liaoning, 110016, P. R. China.

Departments of Diagnostic Radiology, Surgery, Chemical and Biomolecular Engineering, and Biomedical Engineering, Yong Loo Lin School of Medicine and College of Design and Engineering, National University of Singapore, Singapore, 119074, Singapore.

Email: wangkaiyuan@hotmail.com

Y. Tang, X. Lu, H. Tang, and S. Ning

Research Center of Nanomedicine Technology, The Second Affiliated Hospital of Guangxi Medical University, Nanning, 530000, China.

Email: nspdoctor@sr.gxmu.edu.cn

X. Chen

Departments of Diagnostic Radiology, Surgery, Chemical and Biomolecular Engineering, and Biomedical Engineering, Yong Loo Lin School of Medicine and College of Design and Engineering, National University of Singapore, Singapore, 119074, Singapore.

Clinical Imaging Research Centre, Centre for Translational Medicine, Yong Loo Lin School of Medicine, National University of Singapore, Singapore 117599, Singapore.

Nanomedicine Translational Research Program, Yong Loo Lin School of Medicine, National University of Singapore, Singapore 117597, Singapore.

Theranostics Center of Excellence (TCE), Yong Loo Lin School of Medicine, National University of Singapore, 11 Biopolis Way, Helios, Singapore 138667.

Institute of Molecular and Cell Biology, Agency for Science, Technology, and Research (A*STAR), 61 Biopolis Drive, Proteos, Singapore, 138673, Singapore.

Department of Pharmacy and Pharmaceutical Sciences, National University of Singapore, Lower Kent Ridge Road, 4 Science Drive 2, 117544, Singapore.

Emai: chen.shawn@nus.edu.sg

**Experimental Procedures**

**Materials**

DCFH-DA, Singlet Oxygen Sensor Green (SOSG), Indocyanine green (ICG), Chlorin e6 (Ce6),  DSPE-PEG2000, DSPE-PEG2000-ICG, Methylene Blue (MB), Hydroxyphenyl Fluorescein (HPF) and ELISA kit used in this work were purchased from Guangzhou Ruiao Biotechnology Co., Ltd.(China). All of the aqueous solutions were prepared using purified deionized (DI) water purified with a purification system (Direct-Q3, Millipore, USA). The other solvents used in this work were purchased from Sinopharm Chemical Reagent (China) and Shanghai Macklin Biochemical Technology Co., Ltd. (China).

**Preparation and characterization of Cy-BF**

**Instruments**

^1^H nuclear magnetic resonance (NMR) spectra were measured on a Bruker AVANCE 300M fully digital superconducting NMR spectrometer using DMSO-*d*6 as solvents, and tetramethylsilane (TMS; δ = 0 ppm) was chosen as the internal reference. High-resolution mass spectra (HRMS) were obtained on a Bruker ultrafleXtreme mass spectrometer system operated in matrix-assisted laser desorption and ionization–time-offlight (MALDI-TOF) mode. UV-vis spectra and fluorescence spectra were measured at the HORIBA Duetta spectrometer.

**Synthesis of 2,2-difluoro-4,6-dimethyl-1,3,2-dioxoborane (1)**

Under argon protection, 2,4-pentanedione (10 g, 0.1 mol) and boron trifluoride ether (21.2 g, 0.15 mol) were added separately to a dry dichloromethane solution (50 mL), and the reaction was carried out at room temperature for 8 hours. After the reaction is complete, add saturated sodium bicarbonate aqueous solution (100 mL), extract 2-3 times with dichloromethane solution (100 mL), dry the organic phase with anhydrous sodium sulfate, and remove the organic solvent by rotary evaporation to obtain white solid 1 (13.9 g, yield 94%).

**Synthesis of 1,1,2,3-tetramethyl-1H-benzo [e] indole-3-iodide salt (2)**

Add 1,1,2-trimethyl-1H-benzo [e] indole (2.0 g, 9.6 mmol) and iodomethane (8.9 g, 63.0 mmol) into a pressure vessel, add 10 mL of acetonitrile solvent and react at 50℃. Heat the mixture to reflux overnight. When the solution is cooled to room temperature. Then slowly add ethyl acetate (100 mL). The precipitate was collected by filtration and washed with ethyl acetate. Collect the crude product and reflux with ethyl acetate (100 mL) for 15 minutes. Cool the suspension to room temperature. The product was collected by filtration and washed with ethyl acetate to obtain a gray solid 1,1,2,3-tetramethyl-1H-benzo [e] indole-3-iodide salt 2 (3.7 g, yield 97%).

**Synthesis of 2-(1,1,3-trimethyl-1,3-dihydro-2H-benzo [e] indole-2-ethyl) acetaldehyde (3)**

Under ice bath conditions, add phosphorus oxychloride (5.25 g, 34 mmol) dropwise to dimethylformamide (10 mL), control the dripping rate to maintain the temperature at 5℃, and then add 1,1,2,3-tetramethyl-1H-benzo [e] indole-3-iodide salt (9.83 g, 28 mmol) dropwise at the same temperature to a solution formed by dissolving 2.5ml of dimethylformamide. After the dropwise addition is complete, keep the temperature at 35℃ and continue the reaction for 40 minutes. Under stirring, pour the reaction solution into 100 g of ice water mixture, add 20% NaOH solution to adjust the pH to about 10-11, extract three times with ether, dry with anhydrous sodium sulfate, and then spin dry with ether to obtain the product (6.55 g, yield 92%).

**Synthesis of Cy-BF (4)**

Dissolve a mixture of compound **1** (60 mg, 0.406 mmol) with compound **3** (0.501 g, 2.02 mmol) and N, N'- dicyclohexylcarbodiimide (DCC, 0.1 g, 0.49 mmol) in 50 mL of toluene and 2 mL of piperidine. Then install a water separator and reflux under nitrogen protection for 7 hours. The color of the solution gradually turns red, and finally turns blue. Evaporate the solvent under reduced pressure and purify the crude product by column chromatography (silica gel, n-hexane: dichloromethane=1: 5) to obtain a blue powder (26.1 mg, 10.5%). ^1^H NMR (300 MHz, DMSO-*d6*) δ 8.33 (t, *J* = 13.3 Hz, 2H), 8.09 (d, *J* = 8.6 Hz, 2H), 7.90 – 7.80 (m, 4H), 7.56 (t, *J* = 7.5 Hz, 2H), 7.38 (d, *J* = 7.7 Hz, 2H), 7.17 (d, *J* = 8.8 Hz, 2H), 5.85 (d, *J* = 13.8 Hz, 2H), 5.67 – 5.58 (m, 3H), 3.40 (s, 6H), 1.98 (s, 12H).


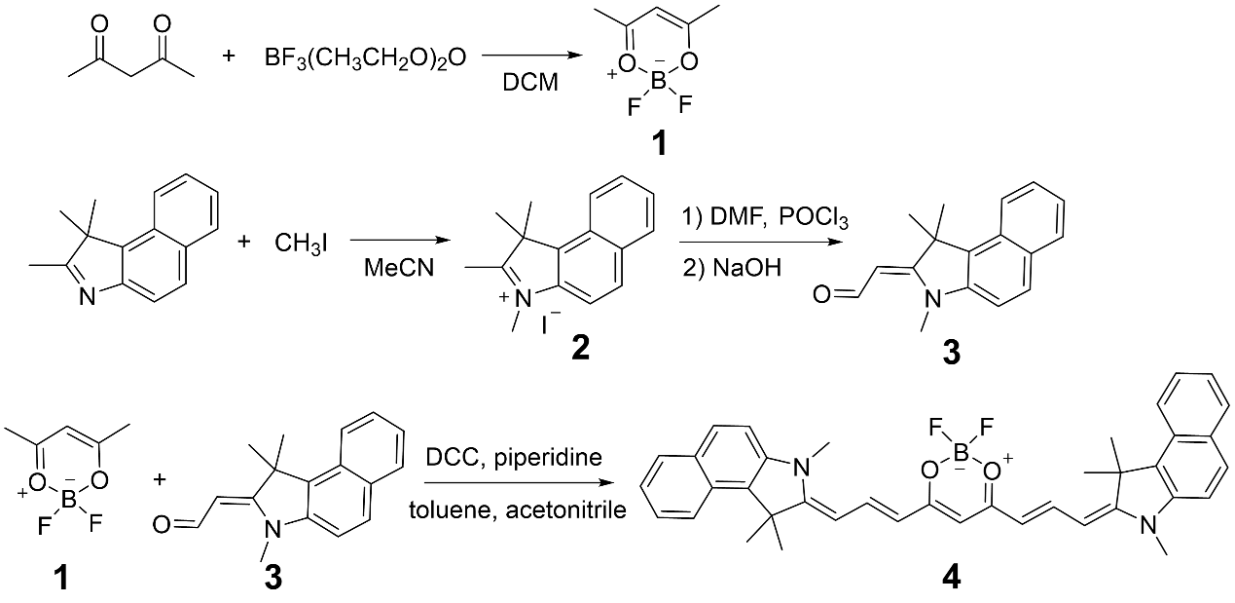


**Scheme S1.** Synthetic routes of Cy-BF.

**Computational details**

The ground-state geometries of molecules were optimized using density functional theory (DFT) method. The polarizable continuum model (PCM), with self-consistent reaction field (SCRF), was used to consider the bulky solvation effects. Time-dependent DFT (TD-DFT) method was utilized at the same level of theory to calculate energy levels of singlet, triplet states and their gap (ΔEST) based on the optimized singlet-state geometries. Analytical frequency calculations were also performed at the same level of theory to confirm that the optimized structures were at a minimum point. Above quantum chemical calculations were carried out by using Gaussian 16 program.

**Preparation and characterization of Cy-BF loaded liposomes (CL), Ce6 loaded liposomes (Ce6-DSPE), ICG loaded liposomes (ICG-DSPE) and platelet exosomes vesicles biomimetic Cy-BF loaded hybrid liposomes (CHL)**

The platelet exosomes vesicles (PEV) derived from BALB/c mice were prepared according to the previous work^[1]^. Then, a mixture of Cy-BF (1 mg), DSPE-PEG2000 (2 mg) and chloroform (1mL) was sonicated (12 W output) to obtain a clear solution. The mixture was quickly injected into 9 mL of water, which was sonicated vigorously in water for 2 min. The mixture was stirred in fume food for 12 h to remove the chloroform. Cy-BF loaded liposomes (CL) suspension was performed for ultrafiltration (molecule weight cut off 100 kDa) at 3000 g for 30 min. Finally, the CL was mixed with 1mg PEV and then repeatedly coextruded through 200 nm pores. The resultant CHL particles were centrifuged and washed with PBS several times to remove the excess PEV. Ce6-DPE was prepared using the same method as CL, except that Cy-BF was replaced with Ce6. ICG-DSPE was prepared using the same method as CL, except that Cy-BF was replaced with 0.1mL chloroform and DSPE-PEG2000 was replaced with DSPE-PEG2000-ICG..

Cy-BF loading capacity were calculated by UV-vis spectra at the UV-vis spectrophotometry Lambda 35 (PerkinElmer). Loading capacity = M_drug_/M_CHL_. where M refers to the mass. The liposomes loaded with Cy-BF (CL) were prepared in the same way, except for removing the PEV. Protein expression was determined by western blot. The particle size and zeta potential were measured by DLS. The morphology of synthesized materials was observed with field-emission TEM (JEM-F200).

**Cell culture**

4T1 mouse breast cancer cell line was obtained from the Cell Bank of the Chinese Academy of Sciences and incubated in RPMI-1640 medium supplemented with 10% FBS in a humidified atmosphere at 37℃.

Cell cultures under normoxic conditions (pO_2_: 21%) were maintained in a humidified incubator at 37℃ in 5% CO_2_ and 95% air. Hypoxic conditions (pO_2_: 2%) were produced by placing cells in a hypoxic incubator (Moriguchi, Japan) in a mixture of 2% O_2_, 5% CO_2_, and 93% N_2_.

**Animal tumor models**

Female BALB/c mice aged 5-6 week were purchased from Vital River Company (Beijing, China). BALB/c mice were subcutaneously injected with 5 × 10^6^ 4T1 cells into the right flank to form tumors. All animal procedures were performed in accordance with the guidelines for Care and Use of Laboratory Animals of the Ministry of Health in People’s Republic of PR China and approved by the Animal Ethics Committee of Guangxi Medical University (Approval number: 2023-KY (0931))**.**

**Photothermal Conversion Efficiency**

A 760 nm NIR laser (Lasever Inc., China) with irradiation powers was used to stimulate the different formulations. The photothermal curve of suspensions during laser irradiation were recorded using an infrared thermal imaging system. The NIR laser source was equipped with a 4 mm diameter laser module with an adjustable power. The photothermal conversion efficiency was calculated using the following equation^[2]^:


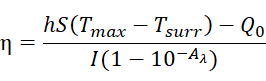


where h is the heat transfer coefficient, S is the surface of the container, T_max_ and T_surr_ are the equilibrium temperature and ambient temperature, respectively. Q_0_ is the heat associated with the light absorbance of the solvent, A_λ_ is the absorbance of CHL at 760 nm, and I is the laser power density.

**Detection of ROS**

SOSG, HPF, and DCF were employed to estimate the generation of ^1^O_2_, •OH and total ROS under NIR laser irradiation (0.5 W/cm^2^, 2 min). The concentration of Cy-BF was 0.02 mg/mL. The MB degradation experiment is also used to detect the generation of hydroxyl radicals in CHL. Typically, the CHL (final Cy-BF concentration 20 μg/mL) was mixed with MB (final concentration 5 μg/mL) in PBS buffer solution (pH 7.4). Subsequently, the system was exposed to NIR laser irradiation (0.5 W/cm^2^, 21 min). The absorbance of MB was measured every 3 minutes using a UV-vis spectrophotometer. Electron Paramagnetic Resonance (EPR) experiments were conducted on Bruker EMXplus EPR spectrometer.

***In vitro* cancer targeting study**

4T1 cells were seeded in 24-well plates and cultured for 12 h. Then, 100 μL CL or CHL (20 μg/mL Cy-BF) was added to the medium. Then, the cells were incubated for different time at 37℃ and 5% CO_2_ and washed with PBS three times. The cells were harvested for analysis of Cy-BF content using high-performance liquid chromatography.

**Intracellular ROS, GSH content, lactate (LA) content, mitochondrial membrane potential, apoptosis, and immunogenic cell death (ICD) detection.**

4T1 cells (1.5 × 10^5^ per well) were seeded in a 12-well plate for 12 h. Then the cells were incubated and treated with 4 different groups: (1) PBS+NIR (0.5W/cm^2^, 10 min); (2) CHL; (3) CL+NIR; (4) CHL+NIR. Cy-BF concentration was 100μg/mL. Then, DCFH-DA, Mitochondrial Membrane Potential Assay Kit (with JC-1), Annexin V-FITC/PI Apoptosis Kit were used according to the instruction. The intracellular GSH and LA content was measured by employing a commercial assay kit (Beyotime).

For ICD detection, the cells were washed with PBS three times, fixed with 4% PFA and permeabilized with 0.1% Triton X-100 for 10 min. After washing with PBS three times, the cells were blocked with 10% FBS, and incubated with Anti-Calreticulin antibody or Anti-HMGB1 antibody (Bioss) and fluorescent labeled secondary antibody for 30 min. The cells were washed with PBS three times, then stained with DAPI for 20 min. Finally, the cells were washed with PBS three times and observed using CLSM. Fluorescence intensity was measured by ImageJ software. For quantification of released HMGB1 and ATP in medium, the medium was collected after the cells were treated with materials. Then 20 μL medium was used for ELISA detection or ATP Assay Kit.

***In vitro* anti-cancer effect of CHL and lithium carbonate (LC)**

Typically, 4T1 cells were incubated in six-well plates at 37℃ with 5% CO_2_ for 24 h; afterward, the culture medium was replaced by new culture medium, cells were incubated with 4 different groups at different Cy-BF concentration: (1) PBS+NIR (0.5W/cm^2^, 10 min); (2) CHL; (3) CL+NIR; (4) CHL + NIR. After incubation for another 6 h. Finally, the viability of 4T1 cells was determined by a CCK-8 cell cytotoxicity assay. Subsequently, we used a similar method to evaluate the killing effect of different concentrations of Cy-BF on 4T1 cells and the toxicity of different concentrations of CHL on RAW 264.7 cells.

***In vitro* anti-cancer effect under different oxygen conditions**

4T1 cells were seeded into the 48-well plate (2 × 10^4^ cells per well). After cultured for 12 h, the cell supernatant was displaced with the fresh culture medium and treated with 4 different groups under hypoxia (pO_2_: 2%) or normoxic (pO_2_: 21%) condition: (1) PBS+NIR (0.5W/cm^2^, 10 min); (2) ICG+NIR; (3) ICG-DSPE+NIR and (4) CL+NIR. After incubation for another 6 h. Finally, the viability of 4T1 cells was determined by a CCK-8 cell cytotoxicity assay.

**Transcriptome gene sequencing**

4T1 breast tumor cells were seeded in 6-well plates (1 × 10^6^ cells/well) and cultured overnight. Cells were treated by PBS+NIR or CHL+NIR as in the apoptosis experiment described above. The total RNA was extracted from cells using the TRIzol. Later, the 150 bp double ended sequencing was performed using Illumina Hiseq instrument. All differentially expressed genes between groups were shown in volcano graphs, and specific DEGs were represented by heat maps. Moreover, screening and identification of differentially expressed mRNA for KEGG pathway analysis was carried out.

**Transwell experiment on Bone marrow-derived dendritic cells (BMDCs) stimulation in vitro**

BMDCs were isolated from 8-week-old BALB/c mice bone marrow. For BMDCs maturation assay, 1 × 10^5^ 4T1 cells were treated by the above four groups and then cocultured with 1 × 10^6^ BMDCs in the transwell culture system, and BMDCs were then isolated by anti-CD11c magnetic beads (NovoBiotechnology Co., Ltd.). Then BMDCs were stained with FITC-anti-CD80 and PE-anti-CD86 (Abcam). Finally, the cells were sorted using flow cytometer (Beckman-Coulter, USA). The secretion levels of cytokines including TNF-α, IL-6, and IL-12p70 in the samples were tested with ELISA kits.

**Cell isolation from spleens**

Spleens were aseptically isolated from mice and incubated at 37℃ in 10% FBS RPMI media containing 1.4 mg/ml collagenase A (Roche) and 30 μg/ml DNase I for 60 min. The treated lung tissue and spleen was dissociated over the 70 μm cell strainer (Fisherbrand). Strainer was washed to collect single-cell suspension. Red blood cells were lysed with ACK lysing buffer (Lonza) for 5 min following by washing of cells with culture media. Cells were counted and adjusted to 5 × 10^6^ cells/ml. Spleen cells were washed twice in fresh magnetic-activated cell sorting (MACS) buffer. CD8^+^ T cells were magnetically purified via negative selection using MACS cell separation system according to the manufacturer’s protocols.

**Western blot and immunofluorescence experiments**

Add CD8^+^ T cells to 1640 medium (containing 10% FBS) and adjust the cell density to 2 × 10^6^/mL, while adding 100U/mL IL-2, CD3/CD28 beads, the cells were then spread into a 24 well plate and divided into 6 groups for 24h: (1) PBS; (2) Lactate (10mM); (3) LC (10mM); (4) CHL; (5) CHL+LC; (6) CHL+LC+LA. Cy-BF concentration was 0.1mg/mL. The expression of MCT1, COX IV and VDAC1 was analyzed by western blot and immunofluorescence experiment according to the standard protocol^[3]^.

**T Cells Response Measurements**

BMDCs were generated from bone mesenchymal stem cells harvested from mice and were preseeded in 6-well plates at a density of 1×10^5^ per well and incubated for 24 h. The different formulations (PBS+NIR, CHL or CHL+NIR) were treated with 4T1 cells and then the dead tumor cells were incubated with BMDCs for 24 h. Cy-BF concentration was 0.1mg/mL. Spleens were surgically removed to prepare single-cell suspensions as mentioned above, which were then added into the plates. After incubation for 24 h, 10 mM LA or LC or LA+LC were then added into the plates. After incubation for 24 h, The secretion of IL-2 in the supernatant and expression of IFN-γ and GZMB in CD8^+^ T cells was detected by ELISA and flow cytometry.The upper layer of the medium was added to the plates pre-seeded with 4T1 tumor cells (3×10^4^ per well in 24-well plates), and the concentration of LDH in the supernatant were detected by LDH Assay Kit.

***In vivo* biodistribution study**

Female BALB/c mice aged 5-6 week were purchased from Vital River Company (Beijing, China). BALB/c mice were subcutaneously injected with 5 × 10^6^ 4T1 cells into the right flank. When tumors reached 300 mm^3^, tumor bearing mice (n = 3) received an intravenous (*i.v.*) injection of 100 μL PBS containing DiR labeled CL or CHL (with a Cy-BF dose of 10 mg/kg). Then the mice were sacrificed at different time after injection to collect the tumors and major organs for fluorescence imaging by *In Vivo* Imaging System (IVIS).

***In vivo* anti-tumor study**

Female BALB/c aged 5-6 week were purchased from Vital River Company (Beijing, China). BALB/c mice were subcutaneously injected with 5 × 10^6^ 4T1 cells into the right flank. When the tumor grows to approximately 100 mm^3^, treatment is carried out on day 0. The mice were firstly divided randomly into different groups (Each group included 5 mice): (1) PBS+NIR (0.5 W/cm^2^, 10 min); (2) LC; (3) CHL; (4) CHL+NIR; (5) CL+NIR+LC; (6) CHL+NIR+LC and (7) CL+NIR. The Cy-BF dose was 10 mg/kg. CHL or CL were administered intravenously and undergo phototherapy on day 0. On days 2, 4, 6, 8, 10, and 12, LC was administered orally at a dose of 75 mg/kg each time. Mice body weight and tumor volume in all groups were monitored every 3 days. A caliper was employed to measure the tumor length and tumor width, and the tumor volume was calculated according to following formula. Tumor volume = tumor length × tumor width^2^ / 2. After 18 days of treatment, mice were sacrificed. Five main organs (heart, liver, spleen, lung, and kidney) of all mice were harvested, washed with PBS, and fixed with paraformaldehyde for histology analysis. And the tumor tissues were weighed, and fixed in 4% neutral buffered formalin, processed routinely into paraffin, and sectioned at 4 μm. The primary tumor sections were stained with HE, HMGB1, CRT, TUNEL, Ki-67 and DCFH-DA and finally examined by using fluorescence microscope (IX81, Olympus, Japan). Fluorescence intensity was measured by ImageJ software. The LA content in primary tumor tissues were measured by the Assay Kit (Beyotime Biotech. Inc.). Tumor infiltrating CD8^+^ T cells were isolated using a CD8^+^ T cell sorting kit (Thermo Fisher) and their mitochondria were subjected to WB detection.

To examine DC maturation in vivo, the inguinal lymph nodes (LN) were harvested. The frequency of DC maturation in the LNs was then examined by CD11c^+^ cell sorting kit (Thermo Fisher) and flow cytometry after immunofluorescence staining with FITC-anti-CD80 and APC-anti-CD86 (Biolegend)**.** To study the T cells content and function in tumors, tumors were harvested from mice in different groups and treated with flow cytometry after immunofluorescence staining with FITC-anti-CD3 and PE-anti-CD8 (Abcam); FITC-anti-CD8 and APC-anti-GZMB (Biolegend); PE-anti-IFN-γ and FITC-anti-CD8 antibodies (Guangzhou Ruiao Biotechnology Co., Ltd.). To study the Tregs content in tumors, tumors were harvested from mice in different groups and then examined by CD3^+^ T cell sorting kit (Thermo Fisher). Then the cells were treated with flow cytometry after immunofluorescence staining with PE-anti-Foxp3 antibodies and APC-anti-CD25 antibodies (Biolegend). To analysis treatment-induced cytokine secretion, whole blood was collected from mice at 3 days post first treatment. The serum concentration of proinflammatory cytokines including TNF-α, IL-6 and IFN-γ were then analyzed with ELISA kits (Neobioscience Co., Ltd., China) according to the manufacturer's instructions.

To investigate the effect of exogenous lactate on therapeutic efficacy, we conducted another group of mouse experiments. BALB/c mice were subcutaneously injected with 5 × 10^6^ 4T1 cells into the right flank. When the tumor grows to approximately 100mm^3^, treatment is carried out on day 0. The mice were firstly divided randomly into 4 different groups (Each group included 5 mice): (1) PBS+NIR (0.5W/cm^2^, 10 min); (2) LA; (3) CHL+NIR+LA; (4) CHL+NIR+LC+LA. The Cy-BF dose was 10 mg/kg. CHL was administered intravenously and undergo phototherapy on day 0. On days 2, 4, 6, 8, and 10, LC was administered orally at a dose of 75 mg/kg each time. Inject 1 g/kg LA intraperitoneally every day from day 0 to day 15. Mice body weight and tumor volume in all groups were monitored every 3 days. A caliper was employed to measure the tumor length and tumor width, and the tumor volume was calculated according to following formula. Tumor volume = tumor length × tumor width^2^ / 2. After 15 days of treatment, mice were sacrificed. Five main organs (heart, liver, spleen, lung and kidney) of all mice were harvested, washed with PBS, and fixed with paraformaldehyde for histology analysis. And the tumor tissues were weighed, and fixed in 4% neutral buffered formalin, processed routinely into paraffin, and sectioned at 4 μm. The primary tumor sections were stained with H&E and anti-CD8 antibodies and fluorescence labeled secondary antibody and finally examined by using fluorescence microscope (IX81, Olympus, Japan). Fluorescence intensity was measured by ImageJ software. The LA content in primary tumor tissues were measured by the Assay Kit (Beyotime Biotech. Inc.). The CD8^+^ T cell flow cytometry in tumor tissue was analyzed using the above method.

**Bilateral tumor experiment**

Female BALB/c mice aged 5-6 week were purchased from Vital River Company (Beijing, China). BALB/c mice were subcutaneously injected with 5 × 10^6^ 4T1 cells into the right flank (primary tumors) and 1 × 10^6^ 4T1 cells into the left flank (distant tumors), respectively. The mice were first divided randomly into 6 different groups (Each group included 5 mice): (1) PBS+NIR (0.5 W/cm^2^, 10 min); (2) LC; (3) CHL; (4) CHL+NIR; (5) CL+NIR+LC; (6) CHL+NIR+LC. The Cy-BF dose was 10 mg/kg. CHL or CL were administered intravenously and undergo phototherapy on day 0. On days 2, 4, 6, 8, and 10, LC was administered orally at a dose of 75 mg/kg each time. Mice body weight and tumor volume in all groups were monitored every 3 days. A caliper was employed to measure the tumor length and tumor width, and the tumor volume was calculated according to following formula. Tumor volume = tumor length × tumor width^2^ / 2. After 15 days of treatment, mice were sacrificed. The distant tumor tissues were weighed, and fixed in 4% neutral buffered formalin, processed routinely into paraffin, and sectioned at 4 μm. Then the tumor sections were stained with H&E and anti-CD8 antibodies and fluorescence labeled secondary antibody and finally examined by using fluorescence microscope (IX81, Olympus, Japan). Fluorescence intensity was measured by ImageJ software. The CD8^+^ T cell flow cytometry in tumor tissue was analyzed using the above method. Secretion of pro-inflammatory cytokines (TNF-α and IFN-γ) in sera after different treatments were measured by ELISA.

**Statistical analysis**

Data analyses were conducted using the GraphPad Prism 5.0 software. For variance analysis, One-way analysis of variance (ANOVA) with Tukey’s post hoc test was used. p values of <0.05 were considered significant. *p < 0.05, **p < 0.01, ***p < 0.001.

**References**

[1] Q. Ma, Q. Fan, J. Xu, J. Bai, X. Han, Z. Dong, X. Zhou, Z. Liu, Z. Gu, C. Wang, *Matter* **2020**, *3*, 287-301.

[2] D. Zhu, M. Lyu, Q. Huang, M. Suo, Y. Liu, W. Jiang, Y. Duo, K. Fan, *ACS applied materials & interfaces* **2020**, *12*, 36928-36937.

[3] J. Ma, L. Tang, Y. Tan, J. Xiao, K. Wei, X. Zhang, Y. Ma, S. Tong, J. Chen, N. Zhou, L. Yang, Z. Lei, Y. Li, J. Lv, J. Liu, H. Zhang, K. Tang, Y. Zhang, B. Huang, *Nature immunology* **2024**, *25*, 552-561.

**Supplementary figures**





**Supplementary Fig. 1.** High Resolution Mass Spectrometry (HRMS) of Cy-BF.

**Supplementary Fig. 2.** ^1^H NMR spectrum of Cy-BF.


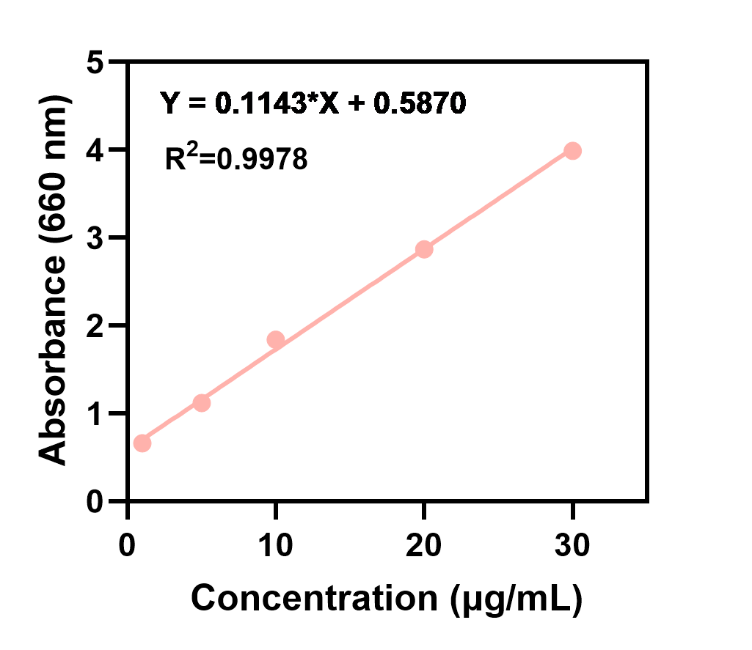


**Supplementary Fig. 3.** The standard curve of Cy-BF in chloroform.


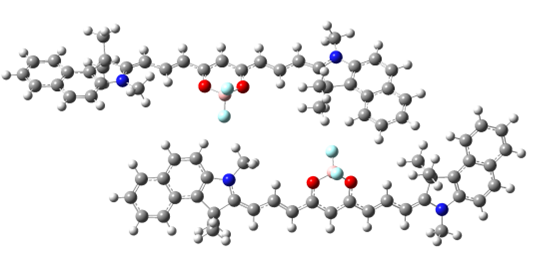


**Supplementary Fig. 4.** Dimeric Cy-BF simulation structure.


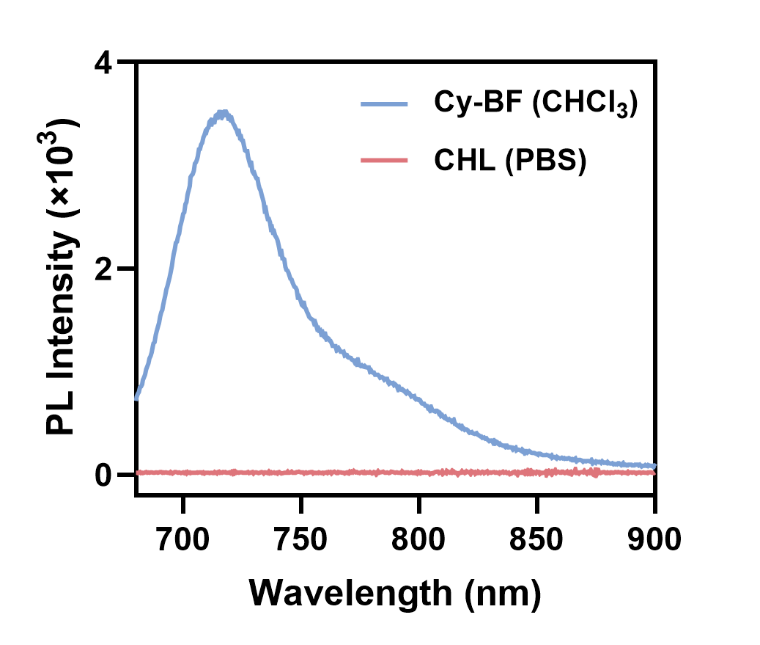


**Supplementary Fig. 5.** Photoluminescence (PL) spectroscopy of Cy-BF CHCl_3_ solution and CHL PBS solution. Excitation wavelength: 660 nm.


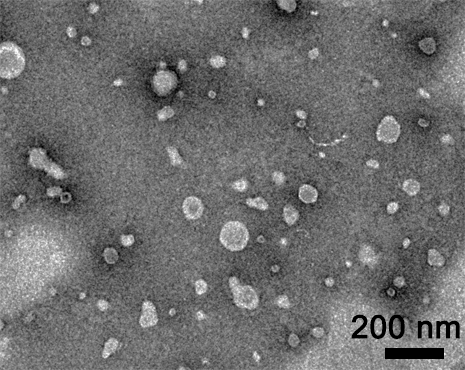


**Supplementary Fig. 6.** TEM image of PEV.


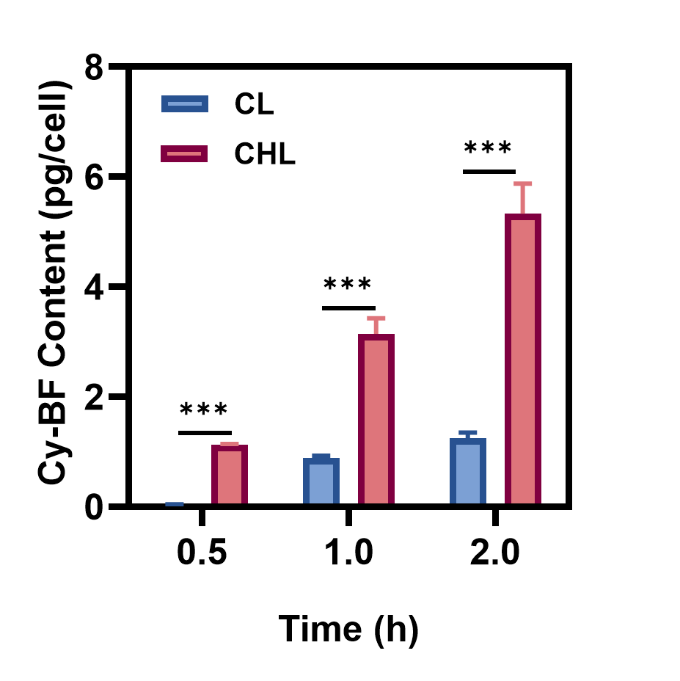


**Supplementary Fig. 7.** Cy-BF content in 4T1 cells at different time points after CL or CHL treatment. Cy-BF: 20 μg/mL, Data are shown as the mean ± SD (n = 3). Statistical significance was calculated via one-way ANOVA with Tukey’s test: ns: Non-Significant, ***p < 0.001.


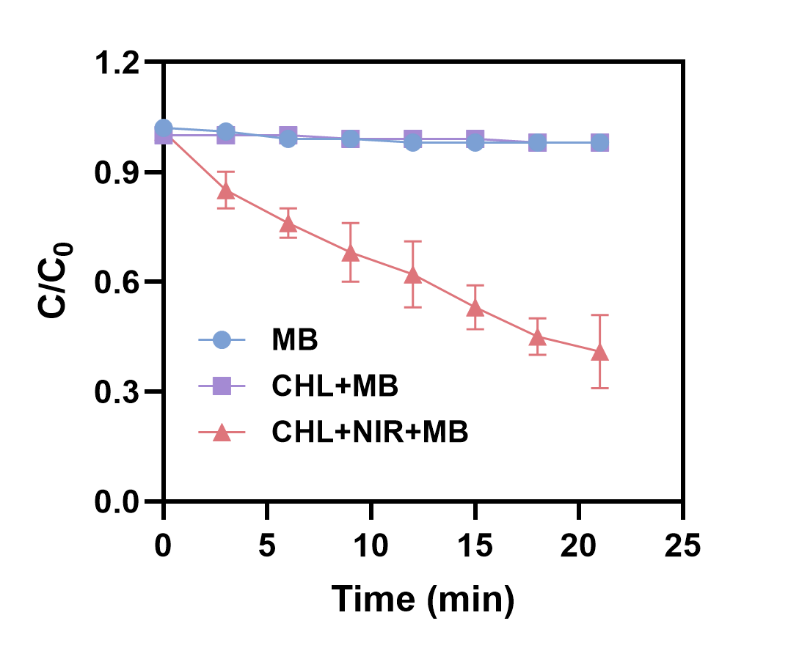


**Supplementary Fig. 8.** Time-dependent MB degradation capacity of different formulations (Cy-BF: 20 μg/mL, MB: 5 μg/mL). Data are shown as the mean ± SD (n = 3).


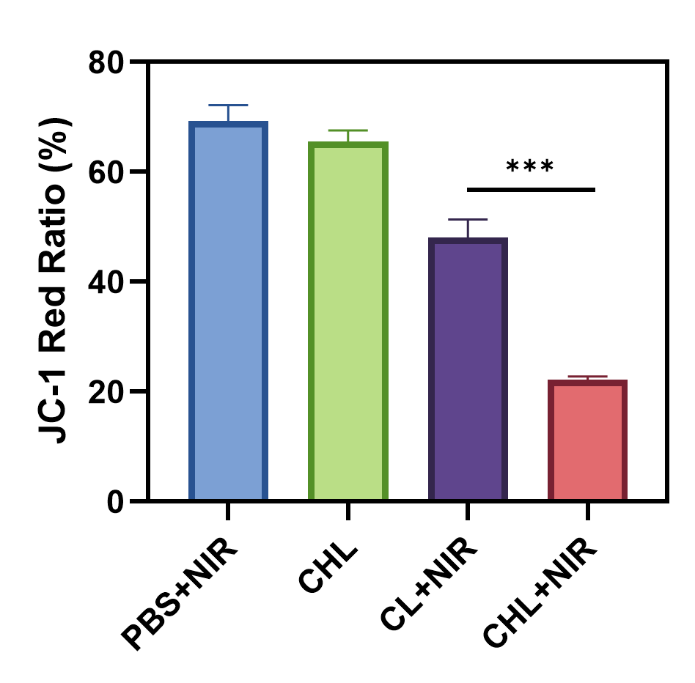


**Supplementary Fig. 9.** Quantification of JC-1 red ratio in Fig. 3A. Data are shown as the mean ± SD (n = 3). Statistical significance was calculated via one-way ANOVA with Tukey’s test: ns: Non-Significant, ***p < 0.001.


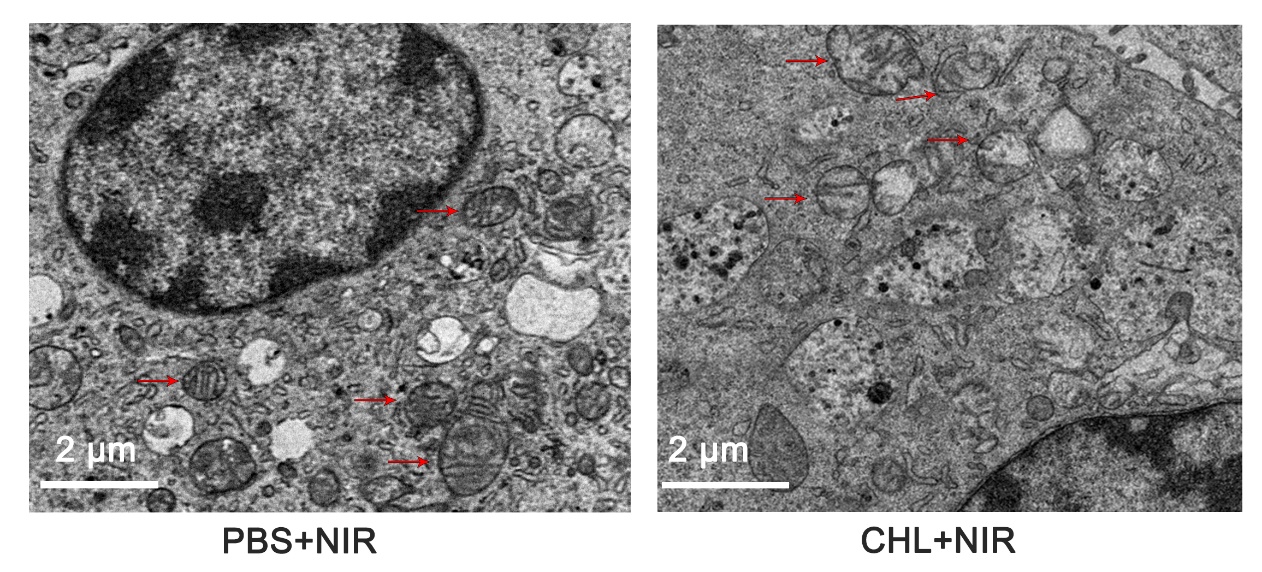


**Supplementary Fig. 10.** TEM images of 4T1 tumor cell after different treatments. The red arrows indicate mitochondria.


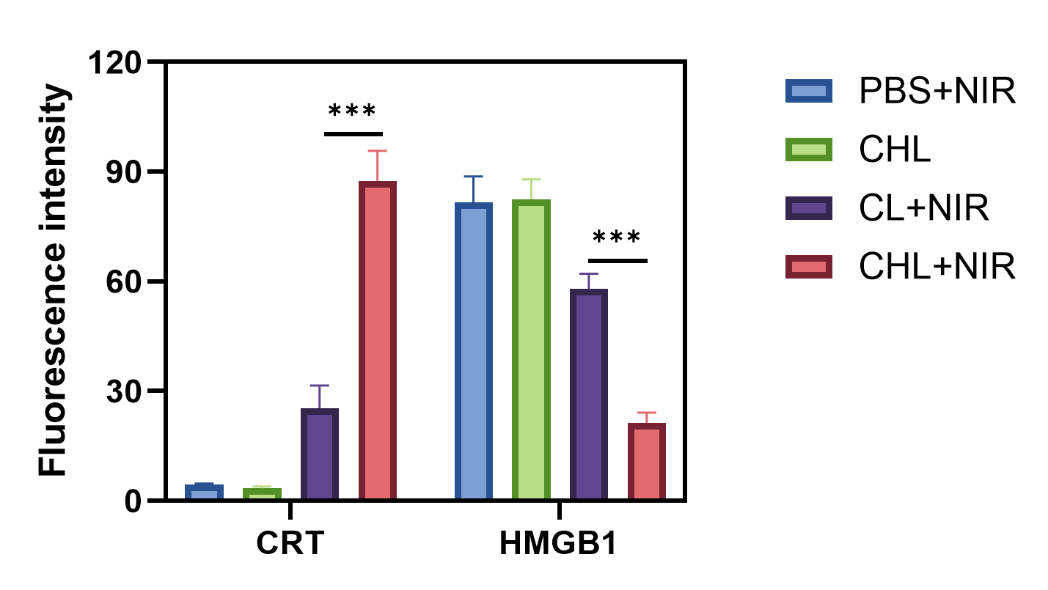


**Supplementary Fig. 11.** CRT and HMGB1 Fluorescence intensity in Fig. 3C were measured by ImageJ software. Data are shown as the mean ± SD (n = 3). Statistical significance was calculated via one-way ANOVA with Tukey’s test: ns: Non-Significant, ***p < 0.001.


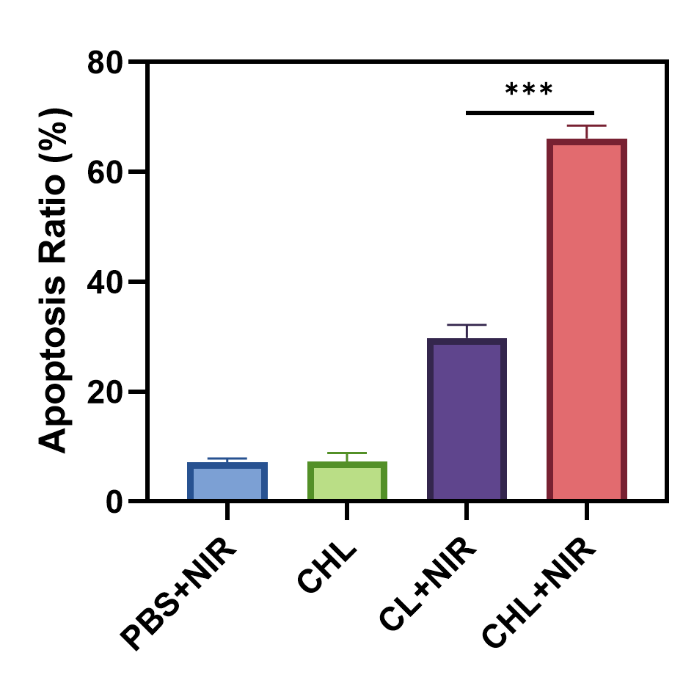


**Supplementary Fig. 12.** Quantification of apoptosis ratio in Fig. 3B. Data are shown as the mean ± SD (n = 3). Statistical significance was calculated via one-way ANOVA with Tukey’s test: ns: Non-Significant, ***p < 0.001.


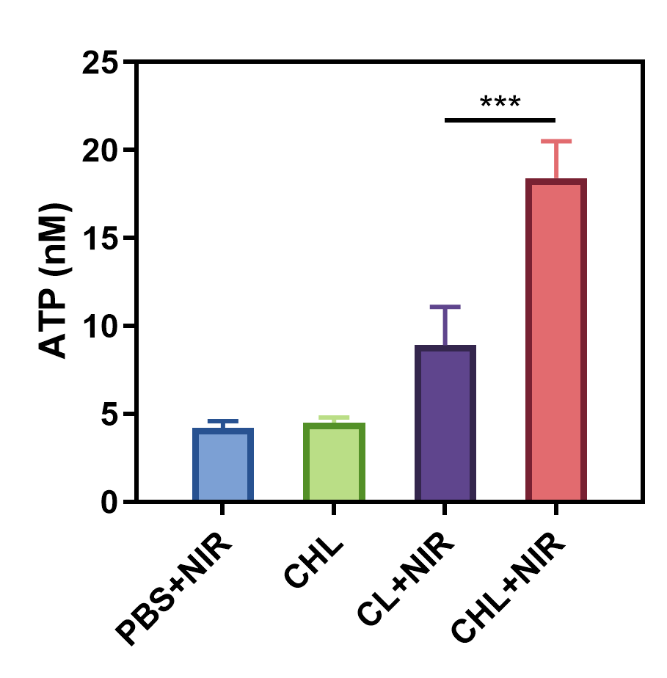


**Supplementary Fig. 13.** Amount of released ATP from 4T1 cells after different treatment. Data are shown as the mean ± SD (n = 3). Statistical significance was calculated via one-way ANOVA with Tukey’s test: ns: Non-Significant, ***p < 0.001.


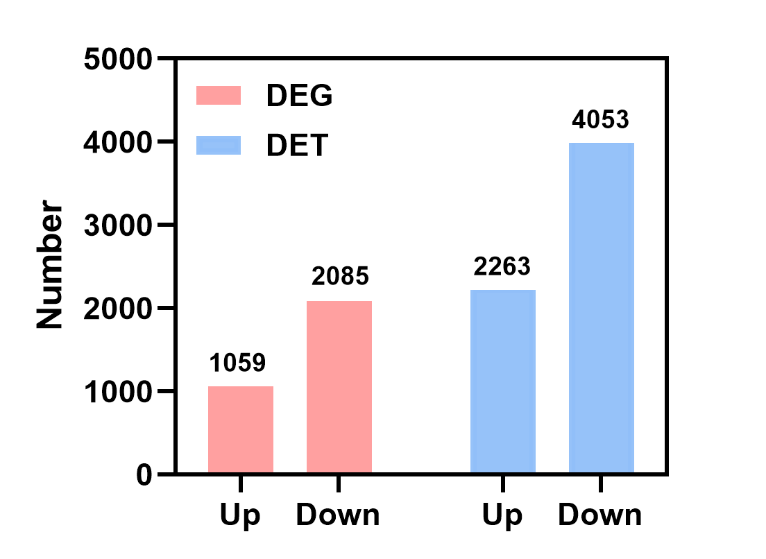


**Supplementary Fig. 14.** Numbers of the down-regulated and up-regulated differentially expressed genes (DEG) and differentially expressed transcripts (DET) of the CHL+NIR group, as compared to PBS+NIR groups.


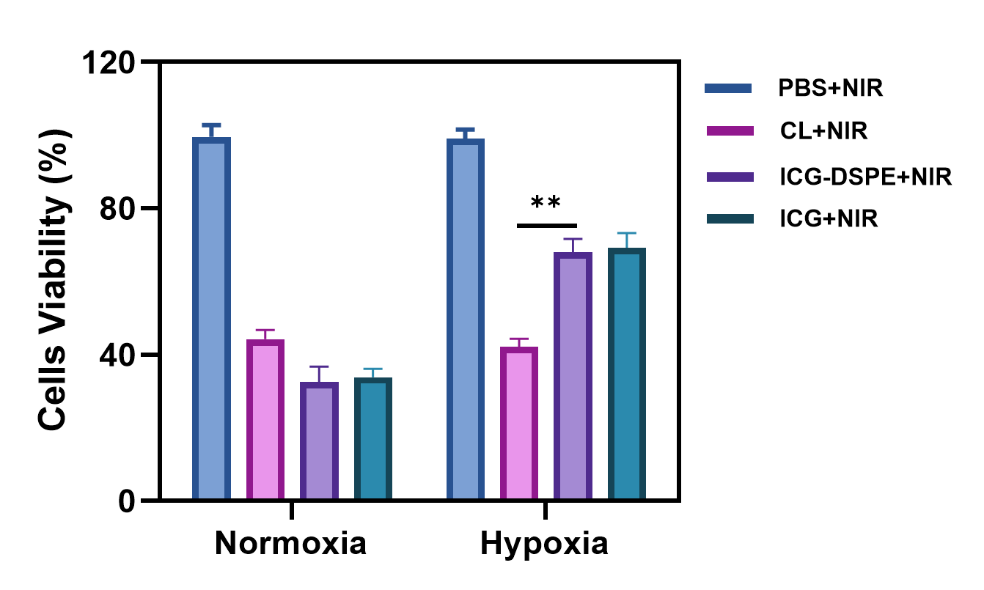


**Supplementary Fig. 15.** The relative cellular viability of 4T1 cells after various treatments under hypoxia or normoxia environment. NIR: 760 nm laser, 0.5 W/cm^2^, 5 min. Data are shown as the mean ± SD (n = 3).


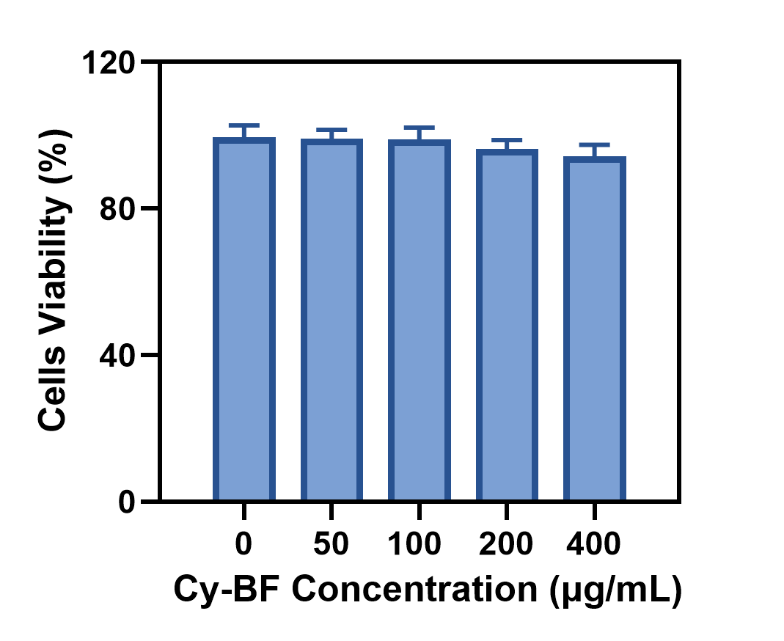


**Supplementary Fig. 16.** The effect of CHL on the survival rate of RAW 264.7 cells with different Cy-BF concentration. Data are shown as the mean ± SD (n = 3).


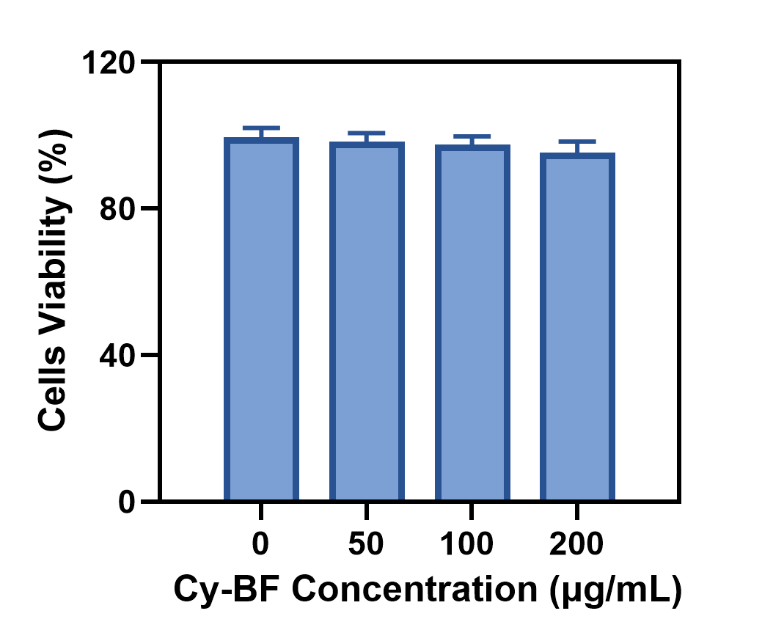


**Supplementary Fig. 17.** The effect of Cy-BF on the survival rate of 4T1 cells with different concentrations. Data are shown as the mean ± SD (n = 3).


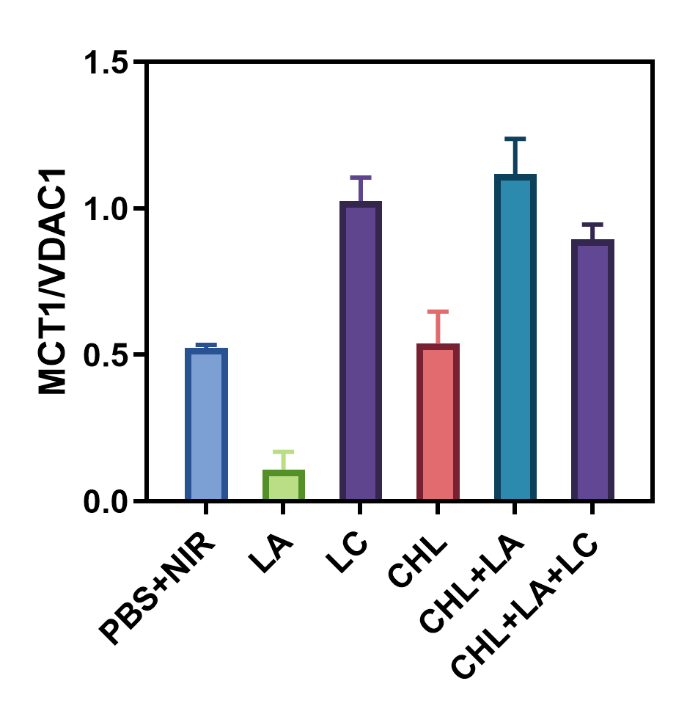


**Supplementary Fig. 18.** Quantification of Western blot analysis for MCT1 *in vitro*. Data are shown as the mean ± SD (n = 3).


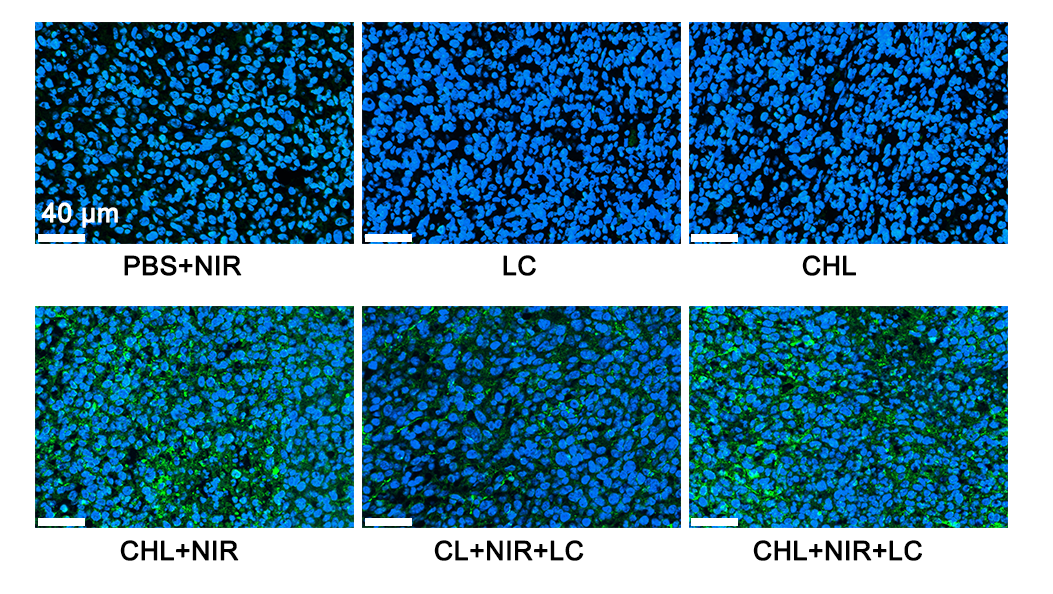


**Supplementary Fig. 19.** ROS staining of primary tumors collected after the indicated treatments.


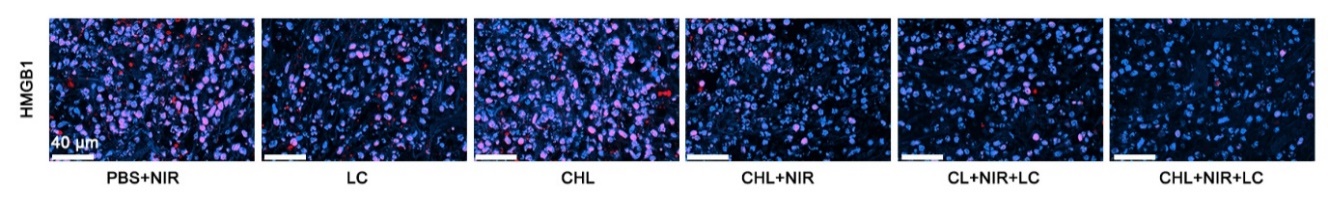


**Supplementary Fig. 20.** HMGB1 staining of tumor sections after the indicated treatments. Blue: DAPI. Red: HMGB1.


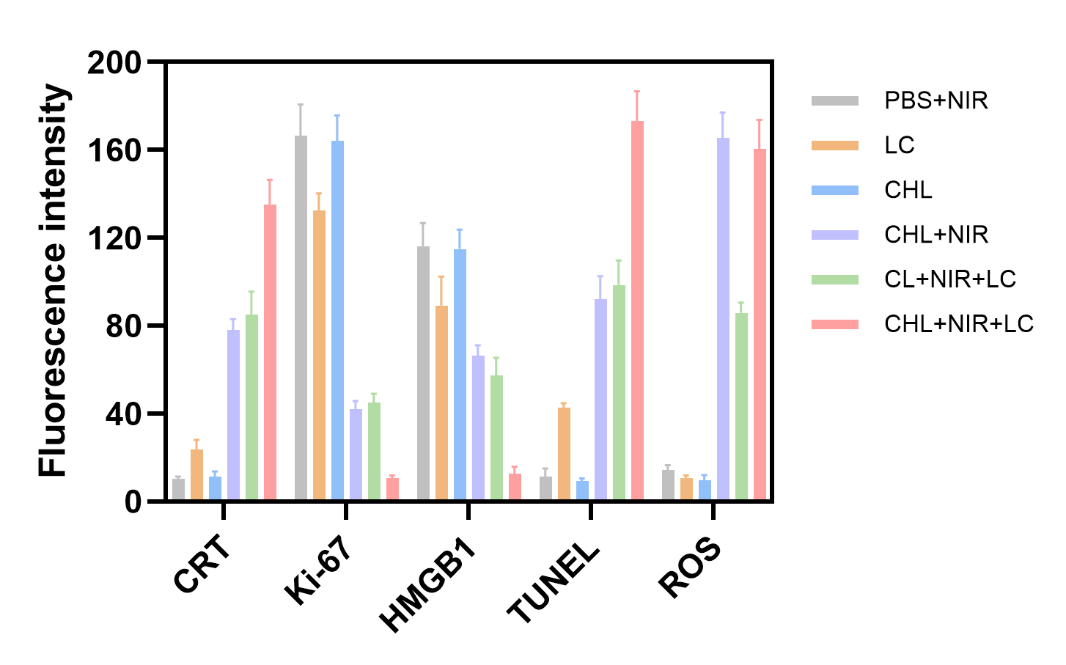


**Supplementary Fig. 21.** Fluorescence intensity in Fig. 6H, Supplementary Fig. 19 and 20 were measured by ImageJ software. Data are shown as the mean ± SD (n = 5).


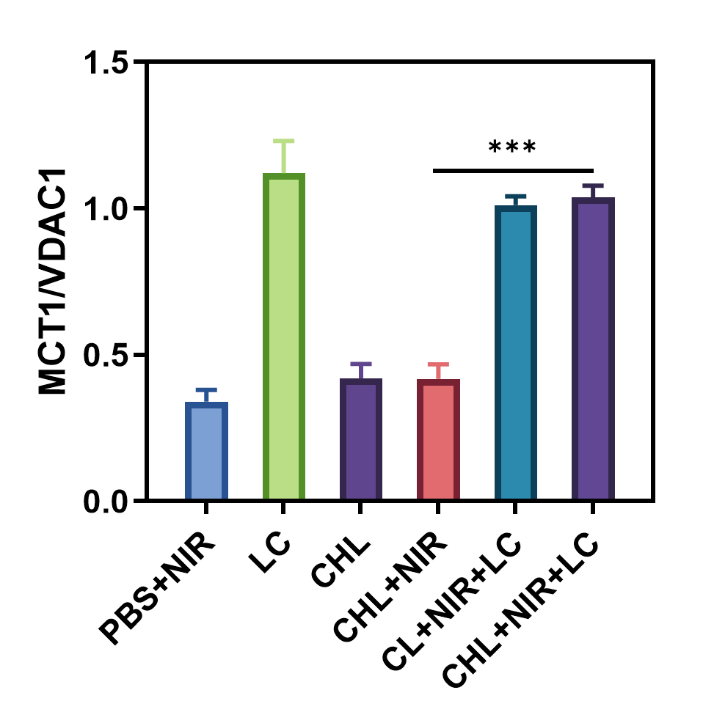


**Supplementary Fig. 22.** Quantification of Western blot analysis for MCT1 *in vivo*. Data are shown as the mean ± SD (n = 5). Statistical significance was calculated via one-way ANOVA with Tukey’s test: ns: Non-Significant, ***p < 0.001.


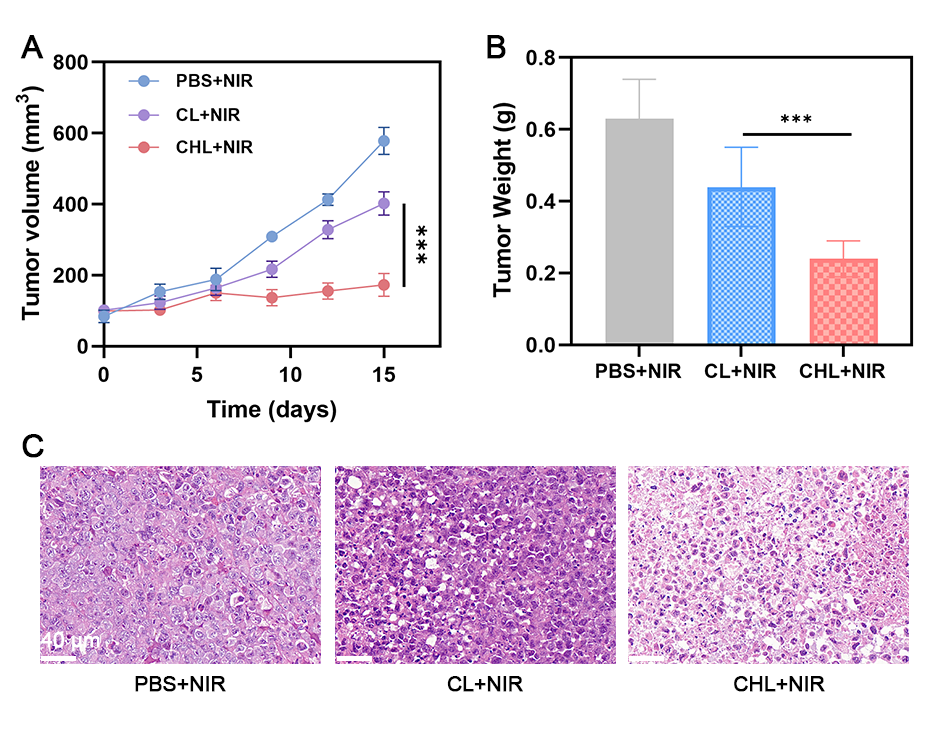


**Supplementary Fig. 23.** (A) Tumor volumes were measured every 3 days after indicated treatments. (B) Tumor weights were recorded f at the end of the study. Data are shown as the mean ± SD (n = 5). Statistical significance was calculated via one-way ANOVA with Tukey’s test: ns: Non-Significant, ***p < 0.001.


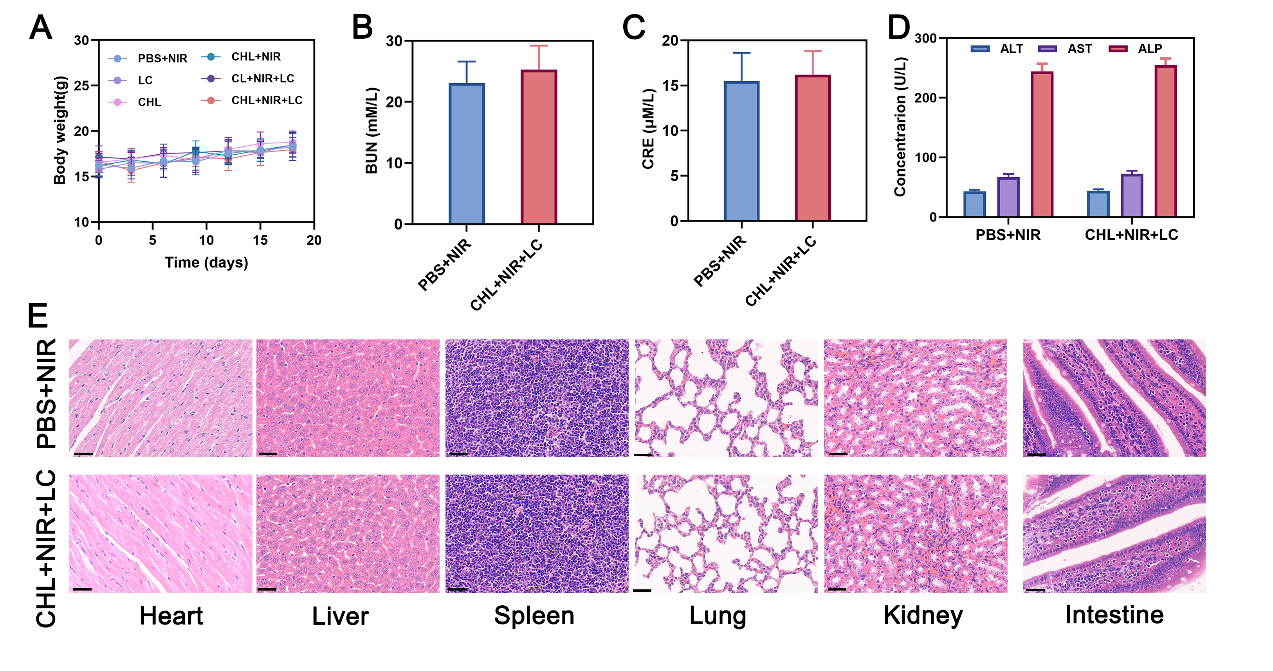


**Supplementary Fig. 24.** (A) Body weight changes during the treatment period. Data are shown as the mean ± SD (n = 5). (B-D) Analysis of liver and kidney function indicators and (E) HE sections of major organs after different treatments. Scale bars: 20 μm. Data are shown as the mean ± SD (n = 5).


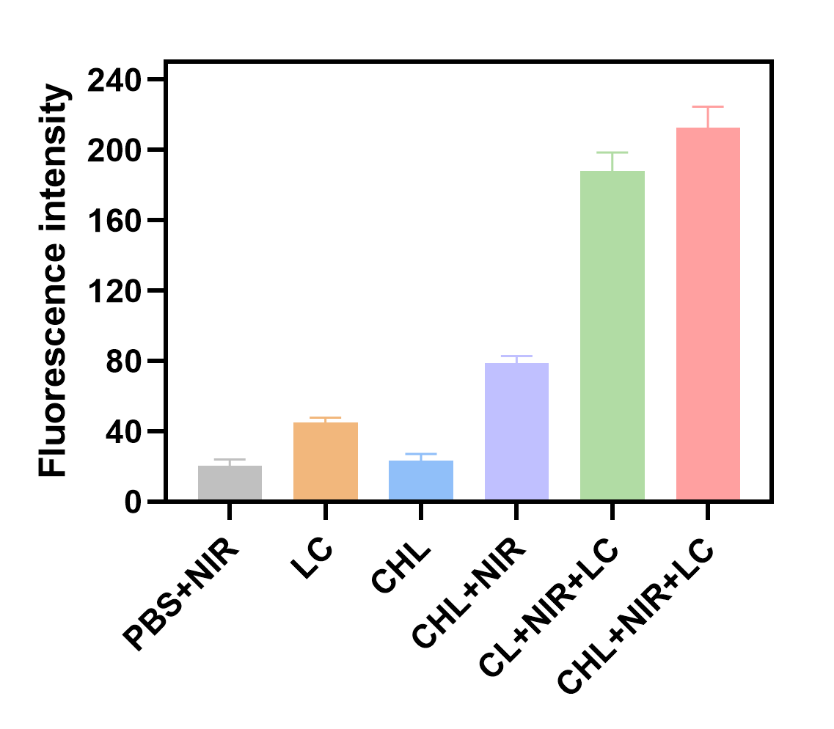


**Supplementary Fig. 25.** CD8 Fluorescence intensity in Fig. 8H were measured by ImageJ software. Data are shown as the mean ± SD (n = 5)


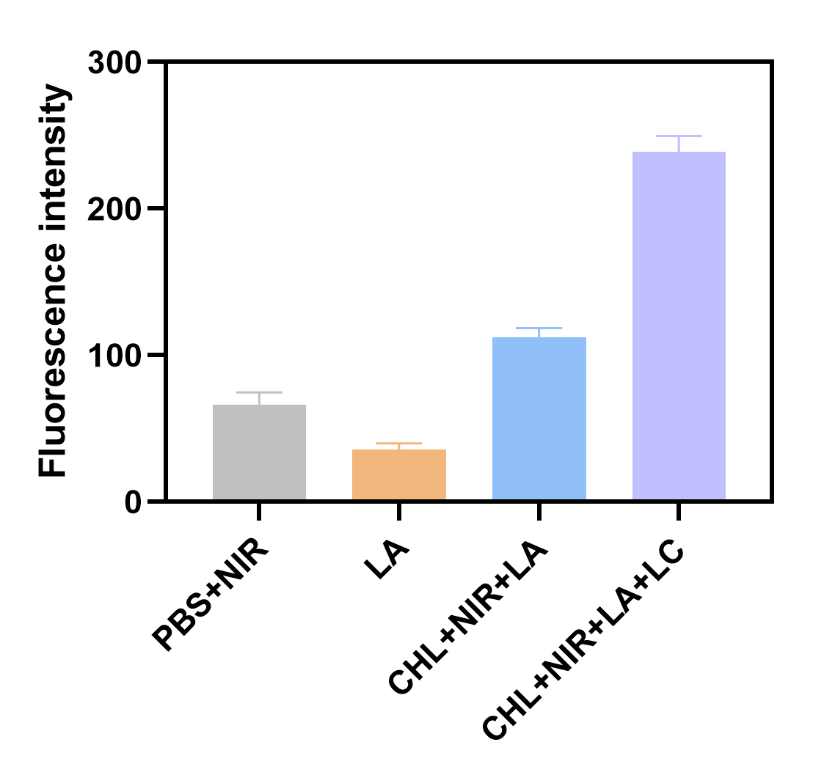


**Supplementary Fig. 26.** CD8 Fluorescence intensity in Fig. 8Q were measured by ImageJ software. Data are shown as the mean ± SD (n = 5)
